# Experimental aortic aneurysm severity and growth depend on topical elastase concentration and lysyl oxidase inhibition

**DOI:** 10.1038/s41598-021-04089-8

**Published:** 2022-01-07

**Authors:** Alycia G. Berman, Daniel J. Romary, Katherine E. Kerr, Natalyn E. Gorazd, Morgan M. Wigand, Sourav S. Patnaik, Ender A. Finol, Abigail D. Cox, Craig J. Goergen

**Affiliations:** 1 Weldon School of Biomedical Engineering, Purdue University, 206 S. Martin Jischke Drive, West Lafayette, IN 47907 USA; 2 Department of Mechanical Engineering, University of Texas at San Antonio, San Antonio, TX USA; 3 Department of Comparative Pathobiology, Purdue University, West Lafayette, IN USA; 4 Purdue Center for Cancer Research, Purdue University, West Lafayette, IN USA

**Keywords:** Vascular diseases, Aneurysm, Experimental models of disease, Three-dimensional imaging, Ultrasonography, Biomedical engineering

## Abstract

Abdominal aortic aneurysm (AAA) formation and expansion is highly complex and multifactorial, and the improvement of animal models is an important step to enhance our understanding of AAA pathophysiology. In this study, we explore our ability to influence aneurysm growth in a topical elastase plus β-Aminopropionitrile (BAPN) mouse model by varying elastase concentration and by altering the cross-linking capability of the tissue. To do so, we assess both chronic and acute effects of elastase concentration using volumetric ultrasound. Our results suggest that the applied elastase concentration affects initial elastin degradation, as well as long-term vessel expansion. Additionally, we assessed the effects of BAPN by (1) removing it to restore the cross-linking capability of tissue after aneurysm formation and (2) adding it to animals with stable aneurysms to interrupt cross-linking. These results demonstrate that, even after aneurysm formation, lysyl oxidase inhibition remains necessary for continued expansion. Removing BAPN reduces the aneurysm growth rate to near zero, resulting in a stable aneurysm. In contrast, adding BAPN causes a stable aneurysm to expand. Altogether, these results demonstrate the ability of elastase concentration and BAPN to modulate aneurysm growth rate and severity. The findings open several new areas of investigation in a murine model that mimics many aspects of human AAA.

## Introduction

An abdominal aortic aneurysm (AAA) is a pathological dilation of the abdominal aorta defined by a 50% increase in diameter or a diameter greater than 3 cm in humans^1^. While aneurysm severity is often defined based on measures of diameter and growth rate, a large variance in patient-to-patient outcomes suggests a complex disease pathophysiology. Broadly, aneurysm progression includes proteolytic degradation of the extracellular matrix (ECM), smooth muscle cell apoptosis, and chronic inflammation of the aortic wall^2^, though the relative contributions of each remains unclear. To further confound factors, 70–80% of patients exhibit intraluminal thrombus (ILT) within their aneurysms^3,4^, but the role of thrombus is still controversial. Given this multifactorial nature of AAA progression and rupture, developing appropriate clinical metrics and new therapies is difficult.

While human studies have provided a wealth of information regarding AAA progression, they are limited as baseline data is often absent and controlled manipulation of experimental variables can be difficult. To this end, experimental animal aneurysms have been used to recapitulate aspects of the human condition^5^. One common model is the elastase perfusion model, which was initially developed in rats^6^ and later optimized for mice^7^. In this model, porcine pancreatic elastase is pressure-perfused into the aorta, which causes an immediate increase in diameter due to the inflation pressure and typically results in an aneurysm by the second week. Bhamidipati et al*.* adapted the elastase perfusion model by applying peri-adventitial elastase, avoiding the need for cannulation of the aorta^8^. Although this model does form aneurysms, they typically stabilize by day 14^9,10^, making it difficult to assess growth dynamics.

Recently, Lu et al*.* combined this topical elastase surgery with continuous oral administration of β-Aminopropionitrile (BAPN), a lysyl oxidase (LOX) inhibitor, to create continuously expanding aneurysms^10^. LOX is produced by vascular smooth muscle cells and is the primary enzyme involved in the formation and maturation of irreducible cross-links in collagen and elastin^11^. By inhibiting LOX and reducing collagen and elastin cross-linking, elastase-treated vessels were unable to stabilize, leading to progressive damage and expansion of the arterial wall, thus creating large aneurysms. In addition, Lu and colleagues noted the presence of ILT in some animals, which is of particular interest given the small number of murine aneurysm models that develop ILT, despite its prevalence in many AAA patients.

Given the ability of combined BAPN and topical elastase to recapitulate continuous infrarenal AAA growth and development of ILT, we utilized this model to further explore the role of elastin degradation and cross-linking on continued aortic expansion. By utilizing high frequency volumetric ultrasound to acutely and longitudinally assess the development of aneurysms, we demonstrate that vessel expansion varies with elastase concentration. Further, by altering the timeline of BAPN application, we show that, while initial elastase degradation strongly influences final aneurysm size and presence of ILT, LOX inhibition also remains necessary for continued aneurysm expansion.

## Methods

### General experimental setup

**Figure 1:**
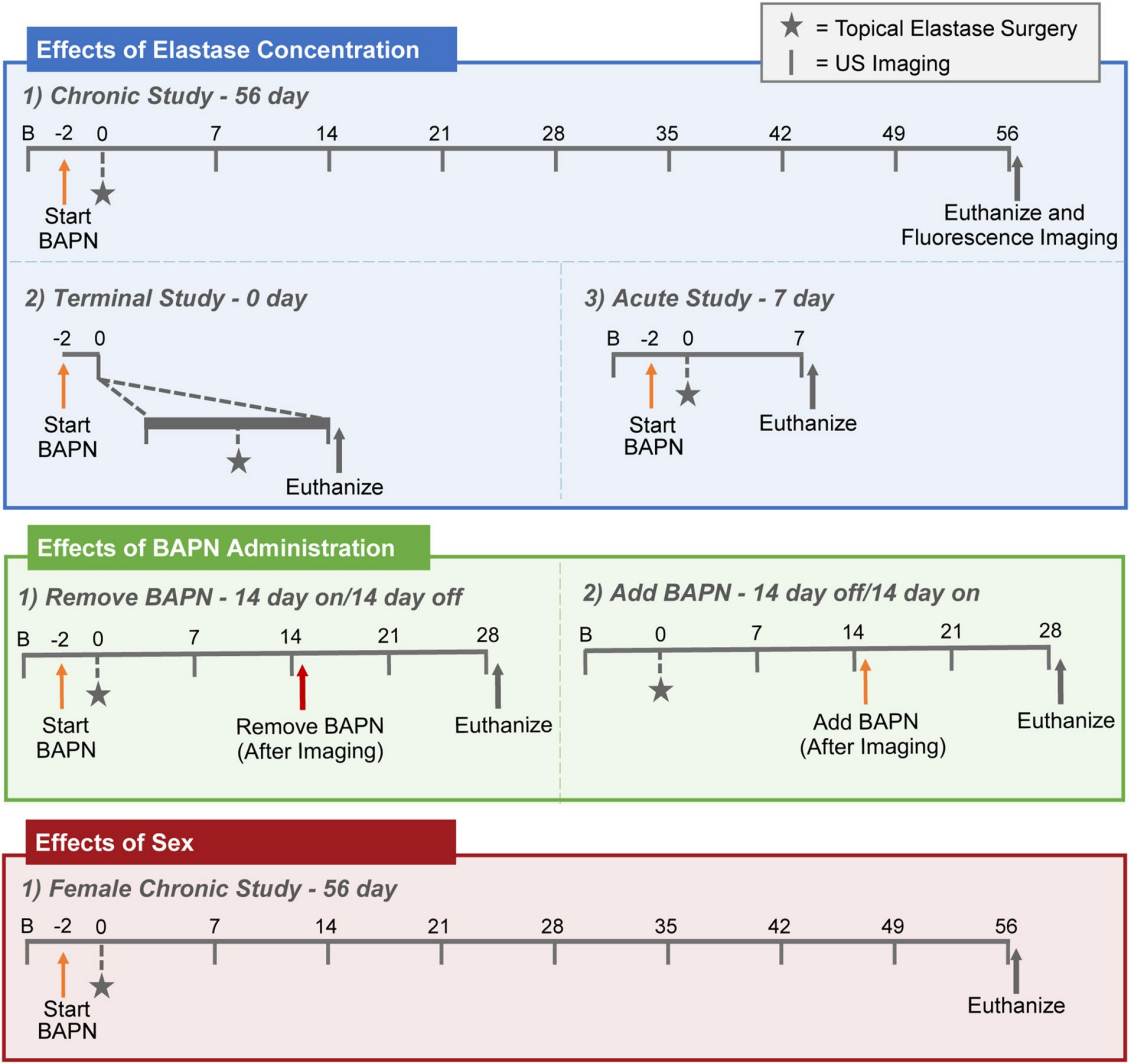
Experimental overview. Schematic depicts the timelines of BAPN administration, elastase surgery, ultrasound imaging, fluorescence imaging, and euthanasia for each sub-experiment.

Data in this study are available on request from the authors. All procedures were performed with approval from the Purdue University Institutional Animal Care and Use Committee (Protocol 1305000869), in accordance with relevant guidelines and regulations, and in compliance with the ARRIVE guidelines. In addition, prior to performing any procedures, mice were acclimated to the facility for a minimum of 3 days. Similar to a previously published protocol^10^, 10-week-old C57BL/6J mice (Jackson Laboratories, Bar Harbor, ME) were given drinking water with 0.2% BAPN fumarate salt (A3134; Sigma-Aldrich, St. Louis, MO), beginning 2 days before surgery and extending through the duration of the study, unless otherwise noted. The majority of these mice were male as was used previously^9,10^; however, a subset (n = 5) of mice were female to assess effects of sex. All mice then underwent topical elastase surgery^10^. Briefly, mice were anesthetized with 2–3% isoflurane and a laparotomy was performed to expose the infrarenal aorta. After blunt dissection to expose the infrarenal aorta, 5 µl of either 2.5 mg/ml, 5 mg/ml, or 10 mg/ml porcine pancreatic elastase (E7885, Sigma Aldrich, St. Louis, MO) was applied directly on the aortic adventitia via a pipette and allowed to remain for 5 min before triple rinsing with saline^12^. To ensure appropriate location of the elastase, only the section of the aorta that was to receive elastase was bluntly dissected. The psoas muscle and inferior vena cava (IVC) also ensured that the elastase remained on top of the aorta for the entire 5 min. After rinsing, the incision was sutured closed and the mice recovered. To track aneurysm progression, mice were imaged with a high-frequency ultrasound system (Vevo 2100 or Vevo 3100, FUJIFILM VisualSonics, Toronto, ON, Canada) before surgery and then regularly afterward (timeline and imaging details below; Fig. 1). At end-of-study, mice were euthanized by isoflurane overdose and bilateral pneumothorax. Cardiac puncture was then performed on a subset of mice, and the systemic vasculature was flushed with saline, paraformaldehyde, and agarose. All aortas were then fixed in paraformaldehyde for histological analysis (details below). Analysis was performed without blinding. A summary of the experiments is shown schematically in Fig. 1.

### Experiment 1—chronic effects of elastase concentration

During surgery, mice were randomly allocated to receive one of three concentrations of elastase: 2.5, 5, or 10 mg/ml (n = 8/group, based on prior work^10^). Male mice were imaged with ultrasound at baseline, as well as weekly post-surgery. After 8 weeks, mice were euthanized, near infrared (NIR) imaging was performed on explanted aortas (details below), and aortas were subsequently fixed for histology. In addition to the three elastase concentrations, 5 additional mice received elastase that had been inactivated by heating to 100 °C for 30 min (heat-inactivated negative controls) and were imaged at baseline and again at weeks 1, 2, 4, and 8 post-surgery.

### Experiment 2—short-term effects of elastase concentration

Similar to Experiment 1, three concentrations of elastase were used: 2.5, 5, or 10 mg/ml (n = 5/group per timepoint), as well as heat-inactivated elastase as a negative control (n = 5 per timepoint). To assess the surgical effects of elastase administration, mice were imaged immediately after surgery by ultrasound and then euthanized. To assess acute effects, mice were imaged and euthanized 1 week after surgery. At both timepoints, aortic tissue was then fixed for histology.

### Experiment 3—effects of LOX inhibition on aneurysm growth

Surgery was performed on male mice with an elastase concentration of 10 mg/ml (n = 6). Two sub-experiments were performed. In the first, BAPN was added to the animals’ drinking water two weeks after surgery to assess if adding BAPN after the aortic tissue had begun to stabilize would still result in aneurysmal growth. In the second sub-experiment, BAPN was removed from the animals’ drinking water two weeks after surgery to assess if LOX inhibition continued to be necessary even after aneurysm formation. For both sub-experiments, mice were imaged before surgery and weekly after surgery. Four weeks post-surgery (2 weeks after either removing or adding BAPN), mice were euthanized, and aortic tissue fixed for histology. Mice with 10 mg/ml elastase and continuous BAPN administration (n = 6) were used for comparator data.

### Experiment 4—chronic effects in females

To assess the chronic effects of sex on AAA progression, five female mice were treated with 10 mg/ml of elastase concentration. As was done for the chronic male study, female animals were imaged with ultrasound at baseline, as well as weekly post-surgery. After 8 weeks, mice were euthanized and aortic tissue fixed for histology.

### Ultrasound imaging

High frequency ultrasound imaging (Vevo 2100; FUJIFILM VisualSonics) was performed on anesthetized mice (1–3% isoflurane). The mice were placed in supine position on a heated stage, connected to electrodes to measure both cardiac and respiration rates. Depilatory cream (Nair, Church & Dwight, Ewing, NJ) was applied to the abdomen to remove hair prior to imaging. Using a MS550D/MX550D linear transducer (22–50 MHz bandwidth; 40 MHz center frequency, FUJIFILM VisualSonics), long- and short-axis brightness (B-) mode and ECG-gated Kilohertz Visualization (EKV) images of the aorta were acquired, in addition to motion (M-) mode at the proximal, maximum diameter, and distal portions of the infrarenal aorta. Lastly, using a linear step motor, sequential 2D short-axis images were collected to create a 3D ultrasound dataset^13,14^.

Effective maximum diameters of the lumen and outer wall were measured at systole using short-axis images. The perimeter of the vessel was hand-drawn to obtain the cross-sectional area (VevoLab 5.5.0, FUJIFILM VisualSonics), and the effective diameter was back calculated by assuming a circular cross-section. In addition, using the M-mode images at the maximum diameter location, we measured systolic and diastolic diameters in triplicate. Systolic and diastolic diameters were then used to calculate Green–Lagrange circumferential strain^15^:

$${E}_{\theta \theta }=\frac{1}{2}\left[{\left(\frac{{D}_{sys}}{{D}_{dia}}\right)}^{2}-1\right]$$

The 3D datasets were manually segmented utilizing the SimVascular platform^16^ to create volumetric segmentations of the aneurysms.

### NIR imaging for MMP activity

A fluorescent probe was used to measure matrix metalloprotease (MMP) activity in the region of the aneurysm. MMPSense 680 (150 µL; PerkinElmer, Waltham, MA) was injected via tail vein approximately 24 h prior to euthanasia. After harvesting the aorta, the specimen was exposed to NIR light at 625 nm to obtain fluorescent images from both the dorsal and ventral sides. For each image, an intensity ratio was calculated as the average pixel intensity of the aneurysm relative to that of healthy thoracic aortic tissue^15^. More specifically, the mean signal intensity was measured for the AAA, the thoracic aorta, and the background using ImageJ (National Institutes of Health, Bethesda, MD^17^). The intensity ratio was calculated as:

$$Intensity \; Ratio=\frac{{I}_{AAA}-{I}_{Background}}{{I}_{Thoracic}-{I}_{Background}}$$

The intensity ratio was calculated for dorsal and ventral images and then averaged to get a single intensity ratio per animal. A representative schematic showing the analysis method is included in Supplementary Fig. S1.

### Histology and immunohistochemistry

All slides were examined by a board-certified veterinary pathology. Specimens were fixed in 4% paraformaldehyde for at least 24 h. Fixed samples were grossly sectioned in ~ 4 mm increments, embedded in paraffin, thinly sectioned at 4 µm onto charged slides, and incubated at 57 °C for 30 min. Following incubation, slides were deparaffinized. Two slides per sample were stained with hematoxylin and eosin (H&E) and Movat’s Pentachrome (MPC) for standard histological analysis. Microscopic examination of the H&E slides was performed by a board-certified pathologist using a semi-quantitative scoring system (Supplementary Methods). Additionally, MPC stained slides were digitally scanned with a Leica Biosystems Versa scanner (Aperio Technologies, Vista, CA). For the terminal and acute studies, the most severely degraded histological section was annotated using Aperio ImageScope software (v12.4.3.50008) to assess elastin degradation in the terminal and acute studies. If no slice had degradation (such as in the heat-inactivated group), a single slice at the mid-infrarenal region was selected instead. The number of dark pixels in the wall was divided by the total number of pixels as a measure of percent elastin in the wall.

Next, antigen retrieval was performed from separate slides for immunohistochemistry in a decloaking chamber at 95 °C for 20 min with DIVA as the retrieval buffer (n = 3–5 per group). After this, slides were rinsed in Tris EDTA buffer placed on the Biocare intelliPATH™ automatic slide stainer. Tissues underwent two blocking steps, first with 3% hydrogen peroxide for 5 min, then 2.5% normal goat serum for 20 min. Primary antibodies (CD45, BD Pharmingenn Biosciences; and Ly6G, Invitrogen) were applied at a 1:200 dilution for 30 min. Slides were rinsed twice with buffer then applied with the secondary antibody, GoR(Mouse adsorbed) ImmPRESS^®^ HRP, for an additional 30 min. Tissues were rinsed twice with buffer then stained with the DAB chromogen for 5 min. Once complete, tissues were counterstained with hematoxylin, dehydrated, and coverslipped with resinous mounting media. Digitally scanned slides were analyzed using the color deconvolution algorithm in Aperio ImageScope. Macros specific to our staining protocol detected positively labeled immune cells (CD45) and neutrophils (Lys6g). Percent positive pixels in a given stained area are reported.

### Statistical analyses

Analyses were performed in Prism 9.0 (GraphPad Software, CA). Data were checked for normality (Shapiro–Wilk test) and homogeneity of variance (Brown-Forsythe test), and violations were corrected with appropriate transformations. The ultrasound data were analyzed using a two-way repeated measures ANOVA to assess main effects of time and elastase concentration (*p* < 0.05). For the female mice, a two-way repeated measures ANOVA assessed the main effects of time and sex (*p* < 0.05). Within each time point, a post-hoc Tukey HSD was performed to assess differences between groups. In addition, within each group, a post-hoc Dunnett’s test was used to assess significant differences relative to baseline data. MMP activity and histology were analyzed using a one-way ANOVA with a post-hoc Tukey HSD (*p* < 0.05). Correlation of thrombus and MMP activity was assessed using Pearson’s correlation. Lastly, thrombus prevalence was measured using a Fisher’s exact test (*p* < 0.05). All data are plotted as mean ± std dev.

## Results

### Elastase concentration influences aneurysm size and growth, but has minimal effect on strain

**Figure 2:**
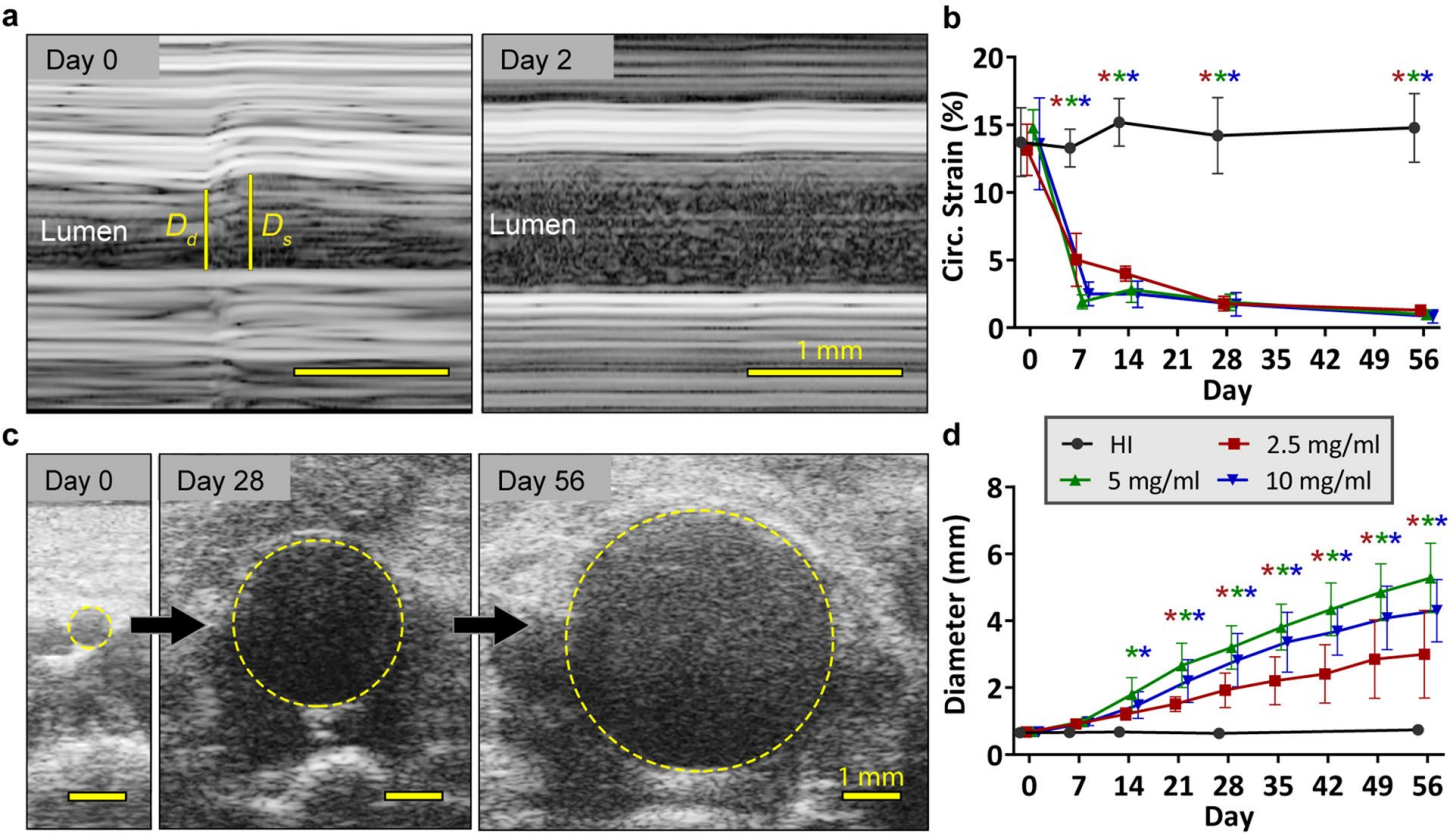
Longitudinal ultrasound imaging of elastase-treated mice. (**a**) Measurements of diastolic and systolic diameters from ultrasound M-mode images were used to calculate Green–Lagrange circumferential strain. (**b**) Post-elastase application, mice showed significantly reduced circumferential strain compared to both baseline measurements and to the heat-inactivated group. In addition, mice treated with 2.5 mg/ml (red) elastase had significantly higher strain than the 5 (green) and 10 mg/ml (blue) groups on day 7. By day 14, measures of strain were indistinguishable among the elastase-treated groups, and remained low for the remainder of the study. (**c**) Short-axis measures of diameter show a continuously increasing aneurysm. (**d**) While all mice had aneurysms, the mice treated with 5 and 10 mg/ml elastase had significantly larger aneurysms than the mice treated with 2.5 mg/ml, beginning at days 21 and 28, respectively. Scale bars are 1 mm. Note that for clarity, the only statistical comparison shown in the graphs are relative to the baseline within each group (*p* < 0.05 with the color of the ‘*’ indicating the group).

**Figure 3:**
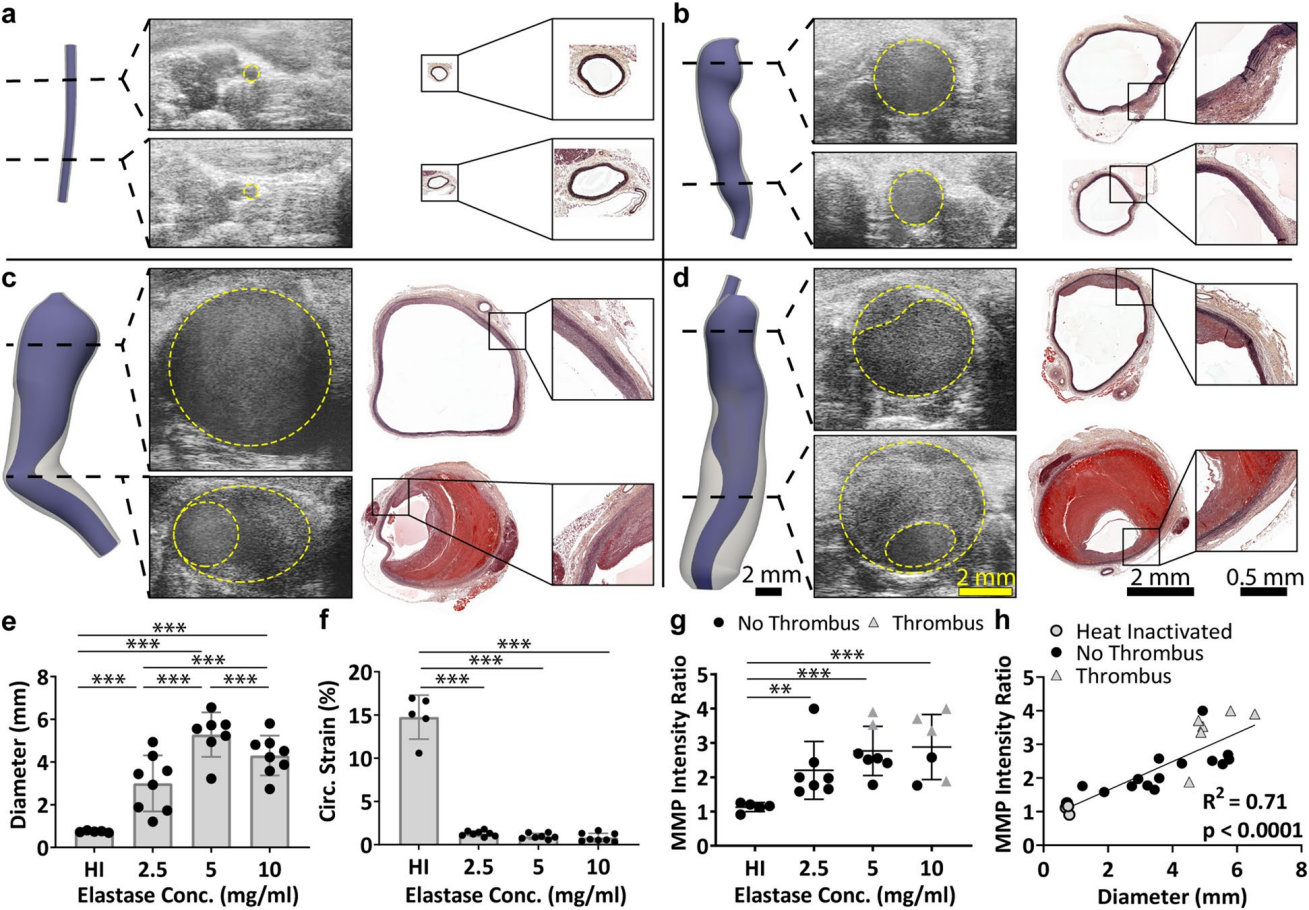
Chronic effects in mice treated with varied elastase concentrations. Ultrasound-based 3D segmentations at day 56 compare well with end-of-study histology (MPC shown). (**a**) In mice treated with heat-inactivated elastase, the aortas showed no degradation of the elastin and remained the same size throughout the study. In contrast, mice treated with (**b**) 2.5 mg/ml, (**c**) 5 mg/ml, and (**d**) 10 mg/ml elastase-treated groups had fragmented elastin and varying-sized aneurysms. In addition, although not all mice in the 5 and 10 mg/ml groups had thrombus, those that did have thrombus tended to have a thin proximal wall and a thrombus-laden distal portion. (**e**) Diameter measurements indicated a graded effect of elastase concentration, with the 2.5 mg/ml group having moderately-sized aneurysms, and the 5 and 10 mg/ml groups having large aneurysms. (**f**) Despite differences in diameter, circumferential strain was statistically indistinguishable among elastase-treated groups, and was only different from the heat-inactivated control. (**g**) MMP activity was also increased in aneurysmal mice, which correlated linearly with diameter (**h**). Of interest, mice with thrombus tended to have higher MMP activity (not statistically assessed). *p < 0.05; **p < 0.01; ***p < 0.001 with lines indicating the comparison.

Following the topical elastase surgery, mice in the 2.5, 5, and 10 mg/ml groups showed reduced strain relative to both baseline values and to the heat-inactivated group, and these strain values continued to remain low for the remainder of the study (*p* < 0.05; Fig. 2a,b). In addition, this reduction in strain was accompanied by a significant increase in diameter, beginning at day 14 for the 5 and 10 mg/ml groups, and at day 21 for the 2.5 mg/ml group (*p* < 0.05; Fig. 2c,d). Of particular interest, elastase concentration clearly influenced final aneurysm size, with the 2.5 mg/ml elastase application resulting in moderately sized aneurysms and the 5 and 10 mg/ml elastase application resulting in significantly larger aneurysms (Fig. 3a–d; Supplementary Figs. S2–S11). By day 56, the diameters of the heat-inactivated, 2.5, 5, and 10 mg/ml groups were 0.74 ± 0.04, 3.0 ± 1.3, 5.3 ± 1.0, and 4.3 ± 0.9 mm, respectively (*p* < 0.01 for all comparisons; Fig. 3e). Interestingly, some of the mice in the 5 mg/ml and 10 mg/ml groups formed thrombus. In the 5 mg/ml group, thrombus was first observed on day 28 and had a final prevalence of 2 in 7 mice (28.6%), while in the 10 mg/ml group, thrombus was first observed between days 28 and 42, and had a final prevalence of 5 of 8 mice (62.5%; *p* = 0.31). While diameter showed a graded effect with elastase concentration, circumferential strain did not. The strain in all three groups was significantly reduced compared to the heat-inactivated group (*p* < 0.001 for all comparisons) but not significantly different from each other (*p* = 0.98 for 2.5 vs 5 mg/ml; *p* = 0.93 for 2.5 vs 10 mg/ml; *p* > 0.99 for 5 vs 10 mg/ml; Fig. 3f).

Further, measures of MMP activity at day 56 were increased in aneurysmal mice and correlated with diameter (*p* < 0.01; Fig. 3g,h). We were unable to obtain MMP data for two of the mice in the 10 mg/ml group due to difficulties with the injections. We also note that one mouse from the 5 mg/ml elastase group in Experiment 1 died on day 42 before the end of the 56-day study and was excluded from all analyses. During necropsy, we observed blood in the abdomen, suggesting aneurysm rupture as the cause of death, although visualization of a rupture site was not possible.

### Elastase application causes immediate reduction in elastin

**Figure 4:**
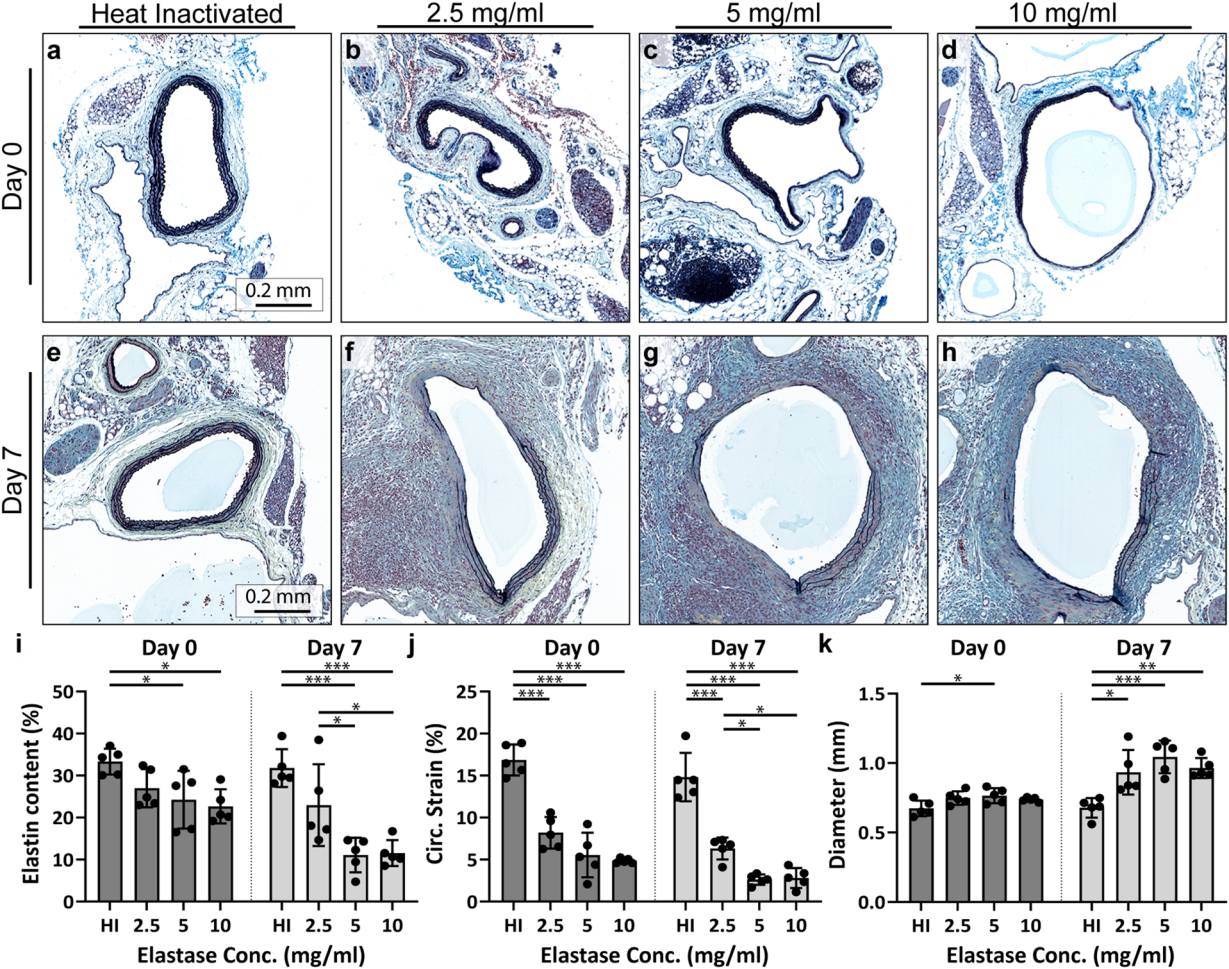
Short-term effects in mice treated with varied elastase concentrations. Terminal and acute MPC and ultrasound results of the topical elastase procedure revealed an immediate reduction in elastin integrity compared to the heat-inactivated group. (**a**) Heat-inactivated elastase-treated mice qualitatively showed no damage post-surgery compared to the (**b**) 2.5 mg/ml, (**c**) 5 mg/ml, and (**d**) 10 mg/ml groups. (**e**) By day 7, heat-inactivated elastase-treated mice similarly showed no damage, but the (**f**) 2.5 mg/ml, (**g**) 5 mg/ml, and (**h**) 10 mg/ml groups also showed diffuse damage. (**i**) Quantification of intact elastin showed similar results. In addition, ultrasound was used to quantify (**j**) strain and (**k**) diameter. *p < 0.05; **p < 0.01; ***p < 0.001.

To assess if the chronic differences observed among the elastase-treated groups were driven by observable acute effects, we performed terminal (euthanize immediately after surgery) and acute (euthanize on day 7) experiments (Fig. 4 and Supplementary Figs. S12–S15). In the terminal group, the 5 and 10 mg/ml elastase-treated mice had an immediate reduction in elastin as quantified with histology (*p* = 0.04 and *p* = 0.01 compared to heat-inactivated, respectively; Fig. 4a–d). The elastin in the 2.5 mg/ml group, although reduced, was not significantly different than that of the heat-inactivated group (*p* = 0.2; Fig. 4i). Noteworthy is that, in all three elastase-treated groups, the destruction of elastin appears heterogeneous, with some regions showing healthy elastin and other regions showing near-complete degradation.

Concomitant with the reduction in elastin content was a reduction in circumferential strain. Immediately post-surgery, all three elastase-treated groups had significantly lower circumferential strain compared to the heat-inactivated mice (*p* < 0.001 for all) but were not significantly different from each other (*p* = 0.16 for 2.5 vs 5 mg/ml; *p* = 0.05 for 2.5 vs 10 mg/ml; *p* = 0.93 for 5 vs 10 mg/ml; Fig. 4j). In addition, diameter was greater in the elastase-treated mice, though it only reached significance in the 5 mg/ml group (*p* = 0.03 vs heat-inactivated; Fig. 4k).

By day 7, much of the outer wall in the elastase-treated aortas lacked clear delineation. Instead, the wall appeared to diffuse into the surrounding tissue (Fig. 4e–h), with mononuclear and polymorphonuclear leukocytes infiltrating into the wall (Supplementary Fig. S16). Per histology, the elastin content in the 5 and 10 mg/ml groups remained low, and were significantly lower than the heat-inactivated group (*p* < 0.001 for both). In addition, by day 7, the elastin content in the 5 and 10 mg/ml groups were also significantly lower than the 2.5 mg/ml group (*p* = 0.03 and *p* = 0.04, respectively). For the 2.5 mg/ml group, although some images showed severe degradation, quantification of elastin suggested that it was non-significantly different than that of the heat-inactivated mice (*p* = 0.13; Fig. 4i). Between days 7 and 56, the elastin in the elastase treated mice continued to degrade, to such a degree that a semi-quantitative scoring by a board-certified pathologist (Supplemental Methods) showed that all mice in the 2.5 mg/ml, 5 mg/ml, and 10 mg/ml groups had the highest score in terms of disorderly arrangement of fibers, disruption in continuity, and distorted form.

Circumferential strain in all three elastase-treated groups remained significantly lower than heat-inactivated (*p* < 0.001; Fig. 4j). In addition, by day 7, the three elastase-treated groups began to differentiate from each other, with the 5 and 10 mg/ml groups showing significantly lower strain than the 2.5 mg/ml group (*p* = 0.02 for both comparisons). Also by day 7, the 2.5, 5, and 10 mg/ml groups had significantly larger diameters than heat-inactivated (*p* = 0.01, *p* < 0.001, and *p* < 0.01, respectively), but were not significantly different from each other (*p* = 0.42 for 2.5 vs 5 mg/ml; *p* = 0.97 for 2.5 vs 10 mg/ml; *p* = 0.67 for 5 vs 10 mg/ml; Fig. 4j).

### LOX inhibition drives continued aneurysm progression

**Figure 5:**
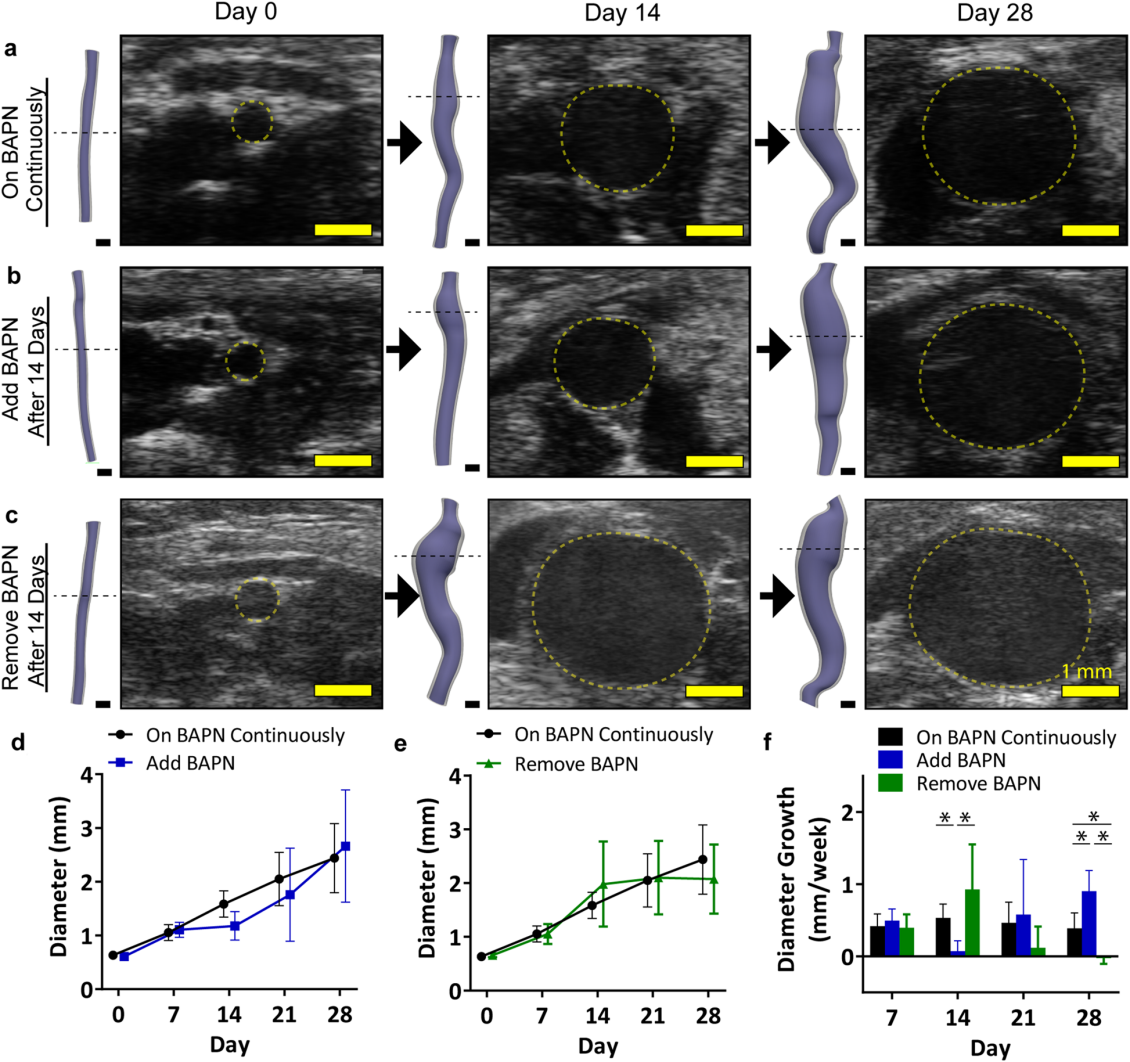
Effects of altering timeline of BAPN administration. Even after aneurysm formation, BAPN administration was necessary for continued aneurysmal expansion. Representative ultrasound and volumetric segmentations demonstrate the lack of growth from Day 14 to Day 28 observed in (**a**) the mice treated with BAPN continuously compared to (**b**) the *Add BAPN* group and (**c**) the *Remove BAPN* group. Quantification of maximum effective diameter demonstrates that (**d**) the addition of BAPN causes growth and (**e**) the removal of BAPN inhibits growth. (**f**) Growth rate shows similar results. Scale bars are 1 mm. *p < 0.05 with lines indicating the comparison.

To probe the effects of BAPN, mice were either (1) given BAPN continuously for 28 days, (2) given regular water for 14 days followed by BAPN for 14 days, or (3) given BAPN water for 14 days followed by regular water for 14 days (Fig. 5a–c). Results demonstrate the necessity of BAPN for aneurysmal growth. Mice given regular water for the first two weeks had aortic diameters that increased from day 0 to 7 (due to the elastase treatment); however, after 7 days, the aortic growth stabilized, resulting in very little growth from day 7 to day 14 (Fig. 5d). Then, after day 14, BAPN was added, and the aneurysms began to expand again.

In a similar manner, the removal of BAPN after aneurysm formation caused stabilization of the aneurysm. Two weeks post-elastase surgery, all mice in the *Remove BAPN* group had aneurysms, with an average maximum diameter of 2.0 ± 0.8 mm (Fig. 5e). However, after BAPN was removed, the aneurysms stabilized and ceased to expand. Two weeks after removing the BAPN treatment, the average diameter growth in the *Remove BAPN* group was reduced to − 0.03 ± 0.08 mm/week (Fig. 5f). In contrast, mice that continued to be treated with BAPN had an average diameter growth of 0.39 ± 0.21 mm/week at the same time point (*p* < 0.01; Fig. 5f).

### Female mice tend to have larger aneurysms.

**Figure 6:**
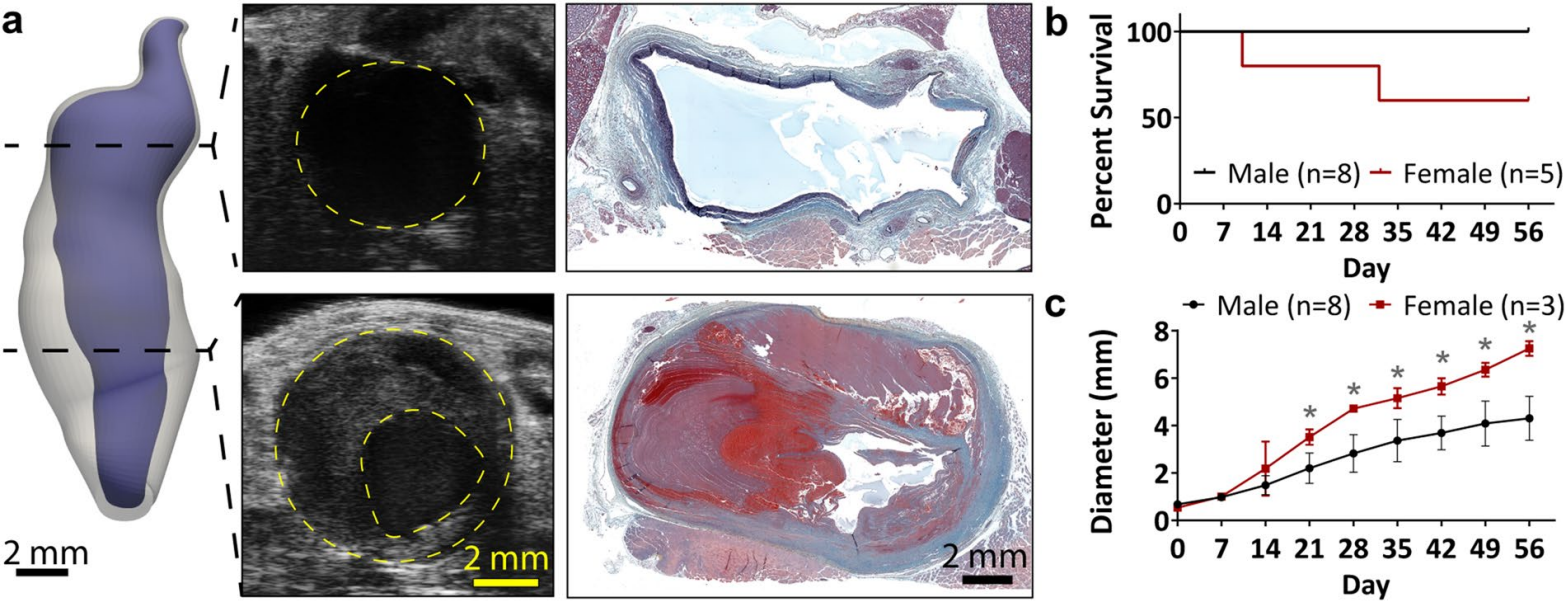
Female elastase-treated mice. Combined treatment of elastase and BAPN in female mice resulted in large aneurysms. (**a**) Similar to the male mice, these aneurysms tended to have a thin, proximal region and a thick, thrombus-laden distal region. (**b**) In addition, two of the five female mice died before end-of-study. (**c**) Among the remaining female mice, maximum diameter was significantly larger than that of the males, beginning on Day 21. *p < 0.05 for male and female comparison within each time point.

Five female mice were treated with 10 mg/ml elastase and BAPN (Fig. 6a and Supplementary Fig. S17), and compared to the eight 10 mg/ml elastase-treated male mice from the concentration study. Of the female mice, two died prior to end-of-study, with one dying on Day 10 and the other dying on Day 32 (Fig. 6b). For the Day 10 mouse, necropsy showed no blood in the abdomen. For the Day 32 mouse, blood was observed in the abdomen, presumably due to aneurysm rupture, although visualization of the location of vessel wall failure was not possible. These two female mice were removed from all subsequent analysis. The three female mice had significantly larger diameters than the male mice, beginning at Day 21 and extending through the rest of the study. At end-of-study, the average maximum diameter of the female mice was 7.3 ± 0.3 mm, compared to average male diameter of 4.3 ± 0.9 mm (*p* < 0.001; Fig. 6c).

### Immune cell infiltration higher in aneurysmal and female mice

The immunohistochemistry analysis revealed substantial immune cell infiltration in the aortic wall in mice that developed AAAs. This was confirmed by applying a broad leukocytic marker (CD45—Supplementary Fig. S18; *p* < 0.05) and more specifically, a marker of neutrophils (Lys6g—Supplementary Fig. S19). In general, a larger percentage of immune cells was observed at day 7 compared to day 56, with more CD45-stained cells compared to Lys6g-positive neutrophils (Supplementary Fig. S20). Interestingly, intermittent LOX inhibition revealed a higher immune cell response in animals where BAPN was given for only the final two weeks compared to the (1) on/off and (2) on continuous groups (*p* < 0.05). Lys6g-positive neutrophil staining revealed a similar trend, although this increase was not significant. Finally, female mice had a non-significant increase in both CD45 and Lys6g stained cells compared to male mice 56 days post-surgery with 10 mg/ml elastase (Supplementary Fig. S20).

## Discussion

The formation and expansion of an AAA is highly complex and multifactorial, and the ability to improve patient care is heavily dependent on increasing our understanding of aneurysm pathophysiology. In this study, we assessed aneurysm growth in a relatively new BAPN + Elastase mouse model^9,10^ by varying elastase concentration and BAPN timing, thus altering the cross-linking capability of the aortic wall tissue. Our results suggest that initial elastin degradation and ultimate vessel expansion is dependent on the concentration of elastase. In addition, we demonstrate that even after aneurysm formation, LOX inhibition remains necessary for continued aneurysmal growth. Namely, removing BAPN, and thus enabling LOX activity, caused stability of the aneurysm. In contrast, adding BAPN caused growth in a previously stable aneurysm. Collectively, these results demonstrate the ability of elastase concentration and BAPN to modulate aneurysm severity.

While the purpose of animal models is to recapitulate aspects of the human condition, no animal model is perfectly able to accomplish this. As such, a myriad of murine models have been developed, each with their own advantages and disadvantages^5,18^. Some of the more established chemical aneurysm animal models include the angiotensin II (AngII) dissection model, the calcium chloride model, and the elastase model. The AngII dissecting AAA model, first described by Daugherty et al.^19^, is created by subcutaneously implanting an AngII-filled mini-osmotic pump into apolipoprotein E-deficient (apoE^−/−^) mice. The resulting aneurysm, which occurs in ~ 60% of the mice, is typically formed due to a tear separating the medial and adventitial layers of the suprarenal aorta^20,21^. The aneurysmal dissection often has both a true and false lumen, with the false lumen filled with intramural thrombus. While there is some growth after the initial onset, it is modest, and aneurysm typically plateaus in size^13,21^. In addition, given its formation and development, the model may better depict an aortic dissection^21^ rather than the fusiform aneurysms that are more commonly observed in the human infrarenal abdominal aorta.

In contrast, both the calcium chloride and elastase models form infrarenal, fusiform aneurysms. The calcium chloride model is formed by topically applying calcium chloride to the adventitial surface of the aorta, causing calcium precipitations that disrupt the elastin and lead to an inflammatory response^22^. However, not all mice form aneurysms and among those that do, maximum diameter remains small, even after 10 weeks^23^. In addition, the chemical burn caused by the treatment is likely not the best representation of the human pathology. Similar to the calcium chloride model, the elastase model can be created by a peri-adventitial application^8^, although initial studies were performed with a more technically challenging intraluminal perfusion procedure^6,7^. Also similar to the calcium chloride model, the resulting aneurysms are small and stable^9^, though prevalence and size are often greater than that of calcium chloride-induced AAAs^7–9^.

While each of these models have their advantages, one common issue is the lack of chronic growth that is typically observed in humans. To counter this, more recent studies have begun to add BAPN to these “classic” models in order to block LOX activity, thus preventing cross-linking of elastin and collagen, and increasing the incidence and/or severity of the disease^9,10,24–28^. In the present work, we used the BAPN + Elastase model, mostly because it resulted in continuously growing aneurysms. Compared to the work of Lu et al., we similarly observed the development of large aneurysms. However, since we concluded our study at Day 56 and not Day 100, we did not observe nearly as many late-stage deaths as they did. We also did not see any early-stage deaths (by Day 8), which may be driven by variations in elastase application and surgical techniques. Building on the work that they had performed, of particular interest to us was the ability to modulate aneurysm severity, which would increase the versatility of this model given the large range of diameters observed in human AAAs^29^. By varying elastase concentration, we were able to modify the maximum aneurysm diameter from ~ 350% increase in the 2.5 mg/ml group to large ~ 500–650% increase in the 5 and 10 mg/ml groups. Presumably, the elastase concentration could be even further reduced to create small but slowly expanding aneurysms.

Although maximum AAA diameter is often used for clinical decisions, 3D imaging and segmentation may provide a more holistic understanding of progression^30^. To this end, we used high frequency ultrasound to obtain 3D datasets from which we created volumetric segmentations of both luminal and adventitial boundaries (Supplementary Figs. S2, S3). One striking feature is the wide variation in shape and degree of tortuosity observed with this model. In general, the proximal portion tended to be larger, potentially driven by differences in mechanics and blood flow in the region near the renal arteries. With that in mind, in an additional experiment, we applied the elastase either closer to the renal arteries or closer to the trifurcation (Supplementary Fig. S21). Although the 3D segmentations suggest an increase in aneurysm size in the mice that received a more proximal elastase treatment, maximum diameter results were highly variable and therefore non-significant (*p* = 0.09 at day 28).

While the proximal portion of the aneurysm tended to be larger, it was the distal portion where we more often observed thrombus formation. Interestingly, this thrombus was intraluminal as has also been noted in human AAAs^31^, and not intramural as is the case in the AngII dissection model. This presence of ILT in a mouse model is noteworthy in that it opens avenues for further exploration of ILT composition, growth, and impact on AAA rupture risk. Such analyses have already begun, with the recent work by Weiss et al*.* who combined histological and biomechanical information in a computational model to provide insight on ILT growth and remodeling^32^.

In the current study, in the 10 mg/ml group, thrombus was observed in 62.5% of the mice, which compares well with the ~ 70% of human cases that develop thrombus^3^ and also matches well with previous data in this model^10^. In contrast, the thrombus frequency in the 5 mg/ml group was only 28.6%. The Fisher exact test was not significantly different between the 5 mg/ml and 10 mg/ml groups and further studies would be required to explore if this slight variation is consistent; however, it may suggest that initial elastase concentration has some effect on eventual ILT formation independent of vessel diameter since both the 5 and 10 mg/ml groups had similar-sized aneurysms. As a simple metric, we found that diameter growth rate in the week prior to thrombus may be a better predictor of initial thrombus deposition than diameter itself (Supplemental Fig. S22). We also observed a highly tortuous region within the non-aneurysmal proximal aorta in some mice that formed thrombus, which then caused a small inflow jet of blood (Supplementary Fig. S23). In addition, some of the thrombus was layered (e.g. M8 in Supplementary Fig. S5) suggesting a staccato process, while other thrombus appeared more homogenous (M1 in Supplementary Fig. S5). Future work utilizing computational fluid dynamic or fluid–structure interaction simulations will be necessary to add mechanistic insight regarding the relationship between flow profiles and ILT. Even so, these preliminary observations provide further support for the idea that flow-induced activation of platelets in the proximal region, followed by stagnant flow in the distal region, may be correlated with ILT formation and warrants further investigation^33–35^.

Tortuosity also varied considerably in the BAPN + Elastase model, with some mice in the 5 and 10 mg/ml elastase groups showing highly tortuous distal sections (Supplementary Fig. S3). Clinically, tortuosity has been associated with increased rupture risk^36,37^ and higher rates of graft-related complications after repair^38^. In our study, increased tortuosity occurred early in some of the mice as also shown previously^9^, but tended to become more exacerbated as aneurysms continued to grow (Supplementary Fig. S2). We also observed that most complex geometries with the highest level of tortuosity were found in the 5 and 10 mg/ml groups, suggesting the vessel was also lengthening as it was expanding radially. This tortuosity seemed to occur independent of thrombus formation, with increased tortuosity noted in both thrombus-laden and non-thrombus-laden aneurysms. Further work will be needed to determine if these severe BAPN + Elastase AAA tortuosities develop in a consistent pattern, as has been previously reported in the AngII dissecting AAA model that commonly expands leftward directly above the right renal artery^15,39^.

Given the difference in chronic aneurysm size among the 2.5, 5, and 10 mg/ml groups, we wanted to explore if these variations were driven by early-stage differences. Indeed, we found an immediate reduction in strain in all three elastase-treated groups compared to the heat-inactivated group. Moreover, quantification of elastin content suggested that the 5 and 10 mg/ml groups had significantly less elastin than the heat-inactivated group, while degradation of elastin in the 2.5 mg/ml group seemed to be more moderate and was not significantly different from either the heat-inactivated group or the 5 and 10 mg/ml groups. Similarly, immunohistochemistry quantification suggests a larger inflammatory response at day 7 compared to 56 for all elastase concentrations. This suggests that even at these early stages, the various elastase concentrations lead to inflammation and differing amounts of elastin degradation, which over the course of 8 weeks, results in varying-sized aneurysms. At day 7, these results are more pronounced, demonstrating a graded effect of elastase concentration.

In addition to understanding the role of elastase concentration, we also studied the impacts of LOX inhibition after aneurysm formation. The importance of LOX in the cardiovascular system has become increasingly apparent^40^. In humans, a LOX missense mutation was discovered in a family with autosomal dominant thoracic aortic dissection^41^. Homozygous mice with the same mutation died of perineonatal aortic aneurysms and hemorrhaging^41^. Similarly, Guo et al*.* found specific rare variants of the LOX gene in humans with enlarged aortic root and ascending aortic dissections^42^. Further study in the smooth muscle cells of humans indicated that in those taken from human AAAs, LOX gene expression was higher, but the LOX functional activity was significantly reduced compared to healthy controls^43^. These all suggest the importance of LOX clinically. However, understanding its role in aneurysm progression is limited. For example, previous work in mice has only assessed the continual presence or absence of BAPN on AAA formation and growth^9,10^. Namely, the question remained: does the aneurysm ever become self-sustaining, whether that be due to hemodynamic, inflammatory, or other reasons? To address this question, we removed BAPN from the animals’ drinking water after an aneurysm had formed by day 14, and noted a near complete stoppage in expansion (Fig. 5c) and reduced inflammation (Supplementary Fig. S20). These results suggest that the influence of hemodynamics and inflammation are secondary to the role of LOX inhibition on continued aneurysmal growth in this animal model.

This influence of BAPN works both ways. Removal of BAPN stabilizes the aneurysm while addition of BAPN can cause growth. In a second sub-experiment, we waited two weeks post-surgery to add BAPN to the water. We chose this time point because previous studies demonstrated that the elastase-only aneurysm stabilizes by day 14^9^. Similar to what was shown previously, there was steady growth between baseline and day 7, followed by minimal expansion from day 7 to 14, suggesting stability. However, after BAPN was added on day 14, the aneurysm began to grow rapidly again and displayed increased inflammatory infiltration. These data demonstrate that LOX inhibition can cause aneurysm growth in previously stabilized AAAs. We do know that this result is not driven by BAPN-induced changes in blood pressure, as mice were normotensive (Supplementary Fig. S24). Instead, we suspect that these effects of BAPN are driven, at least in part, by a reduction of ECM cross-linking^44^ combined with residual inflammation from the elastase treatment^10^, but additional work is needed to fully elucidate this mechanism. It is also likely that the reduction of LOX by BAPN alters the inflammatory response^45^ and impacts endothelial function^40^, both of which are also key contributors to vascular disease. Further work will be needed to assess the specific effects of BAPN and how this impacts aneurysm pathology at both microscopic and macroscopic scales.

A last component of this study was to explore the effects of sex. Clinically, although men have a higher incidence of aneurysm formation, the rupture rate among women is greater than that of men^46–49^. Since these AAA formed by elastase administration which causes severe elastin degradation in all mice, we had hypothesized that both male and female mice would form similar large aneurysms. Both sexes did form large aneurysms; however, the female mice had significantly larger aneurysms with higher inflammatory infiltrate than those of the males at the same time point, suggesting that there may be hormone-specific effects on aneurysmal growth^50^. While this likely does not fully explain the size discrepancies noted, further investigation would be necessary to provide additional mechanistic insight. Such an exploration is beyond the scope of the current study; even so, our results do suggest that there are sex-based differences in this model.

One clear difference of this murine model compared to human pathology is the observation that topical application of elastase caused rapid, severe, and heterogeneous degradation of elastin immediately post-surgery, as demonstrated both by the reduction of in vivo strain and by ex vivo histological/immunohistochemistry analysis. This regional degradation is consistent with the topical (and therefore regional) administration of elastase. While these results were expected given the nature of the surgery, the abrupt change in elastin contrasts with the more gradual reduction that is likely to occur clinically. Clinically, reductions in strain can be indicative of decreased elastin content and increased collagen deposition, which occur during remodeling of the aneurysmal tissue and can result in a stiffer vessel^51^. Unfortunately, once elastin is degraded, it can rarely be recovered, though advances in tissue-engineered solutions may provide avenues that lead to functional elastin deposition and return strain to normal levels. In the current study, while we do observe the concomitant reduction in strain and decreased elastin content, it occurs much more rapidly than would be observed clinically. After the initial abrupt degradation, the vessel remodels and the elastin begin to degrade throughout the vessel, resulting in a disperse degradation at later time points, similar to what has been reported previously^9,10^. However, given this region-specific initial degradation, the BAPN + Elastase model may not be an optimal model for small AAAs or when studying short-term changes. Even so, the relative ease of the surgical procedure, presence of large infrarenal aneurysms, and development of ILT in this BAPN + Elastase model make this a particularly intriguing murine AAA model for future studies.

In conclusion, we demonstrated that elastase concentration and BAPN are able to modulate aneurysm development in this relatively new murine AAA model. Lower concentrations of elastase result in smaller aneurysms, while higher concentrations tend to lead to larger aneurysms. However, this concentration-dependent effect plateaued above 5 mg/ml, with both the 5 and 10 mg/ml groups developing similar aneurysm sizes with ILT formation. In addition, we demonstrated that BAPN could be used to “start” and “stop” aneurysm growth. Thus, elastase concentration and BAPN can be used in tandem to create complex aneurysms that vary in size, growth rate, and presence of ILT. Future work is still needed to elucidate specific reasons for the differences observed, potentially through additional studies of inflammatory cells and markers^52^, other cell types such as smooth muscle cells and endothelial cells^53,54^, production and activation of proteases, and the remodeling of various ECM components such as collagen and glycosaminoglycans^55^. Finally, it is clear that inflammation is a key component in this model as aneurysm growth correlates with inflammatory infiltrate, especially in acute stages of AAA development after considerable elastin damage. Further studies with the BAPN + Elastase model may eventually help in the development of new pharmacological treatments, characterization of the role played by ILT, and improvement in our understanding of how hemodynamics contributes to AAA expansion.

## Supplementary Information

Supplementary Figures.
