# Supplementary material for: Experimental aortic aneurysm severity and growth depend on topical elastase concentration and lysyl oxidase inhibition
Source: Sci Rep. 2022 Jan 7;12:99. doi: 10.1038/s41598-021-04089-8 (PMC8742076; doi:10.1038/s41598-021-04089-8)
Supplement: Supplementary file 1 — Supplementary Figures. [file 41598_2021_4089_MOESM1_ESM.pdf]

Supplemental Material

Supplemental Methods

Histology and Immunohistochemistry

Microscopic examination was performed by a board-certified veterinary pathologist. To evaluate aorta pathology of H&E-stained slides, a semi-quantitative scoring system was applied that assessed four morphological aspects: 1) mononuclear cell infiltrate; 2) polymorphonuclear cell infiltrate; 3) circumferential involvement; and 4) elastin quality. The infiltration of cells was graded on four levels: 0, normal; 1, mild increase in cell numbers; 2, moderate increase in cell numbers; 3, marked increase in cell numbers. The circumferential involvement was graded on four levels: 0, normal; 1, <10% of aorta circumference infiltrated by cells; 2, 10%-50% of aorta circumference; 3, 50% or greater of the aorta circumference. The elastin quality of MPC slides was graded on 4 levels and included qualitative assessment of disorderly arrangement of fibers, disruption in continuity, and/or distorted form. The elastin quality grades were: 0, normal; 1, <25% aorta circumference has distorted elastin; 2, 25%-75% of aorta circumference; 3, 75% or greater of aorta circumference.

For the immunohistochemistry analysis, scanned slides were analyzed using Aperio ImageScope software (v12.4.3.5008) using the color deconvolution algorithm. Macros specific to our staining protocol detected positively labeled immune cells (CD45) and neutrophils (Lys6g). Percent positive pixels in a given stained area are reported for each sub-study (n = 3-5 per group).

Supplemental Figures

MMP Analysis

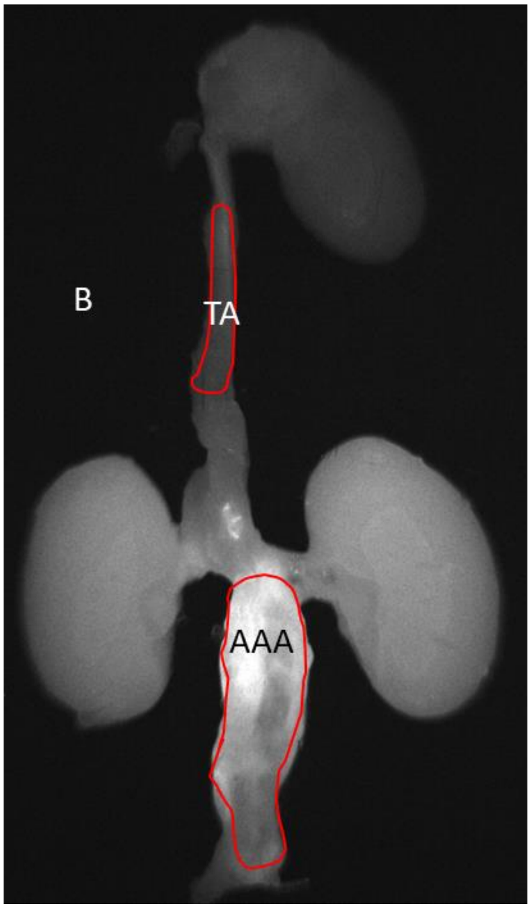

$$Intensity\ Ratio = \frac{\bar{I}_{AAA} - \bar{I}_B}{\bar{I}_{TA} - \bar{I}_B}$$

where I is the average pixel intensity

Supplementary Figure S1 – Representative schematic showing the analysis method used to assess MMP activity. Regions of aneurysmal aorta, thoracic aorta, and background were hand-drawn, and the average pixel intensity calculated for each region. A ratio of aneurysmal intensity to thoracic intensity (after removing the effects of background noise) was used to determine MMP activity.

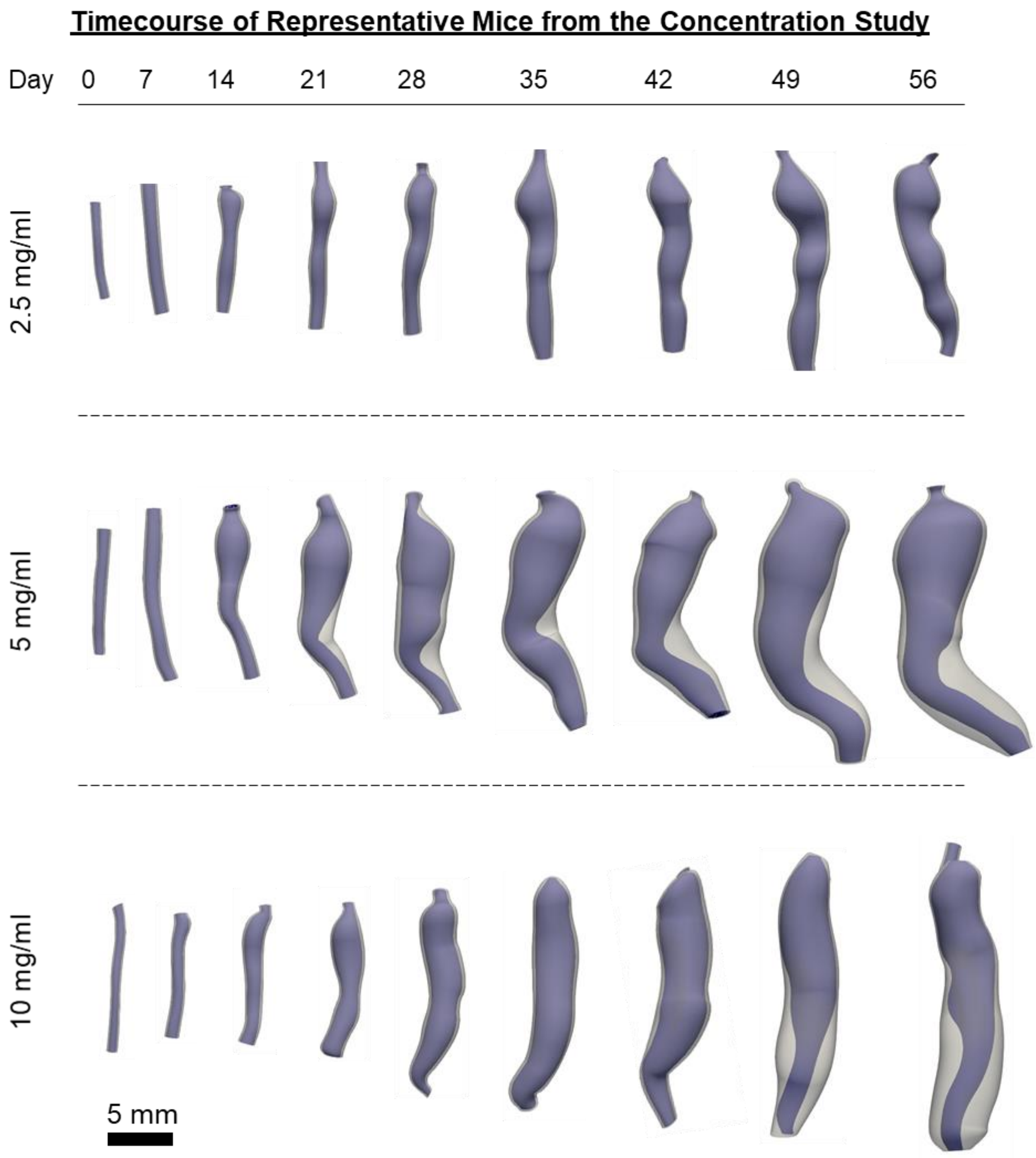

Supplementary Figure S2 – Representative time course for mice treated with 2.5 mg/ml, 5 mg/ml, and 10 mg/ml of elastase. The purple is lumen and the white is outer wall. Locations of thick wall grey in color indicate thrombus.

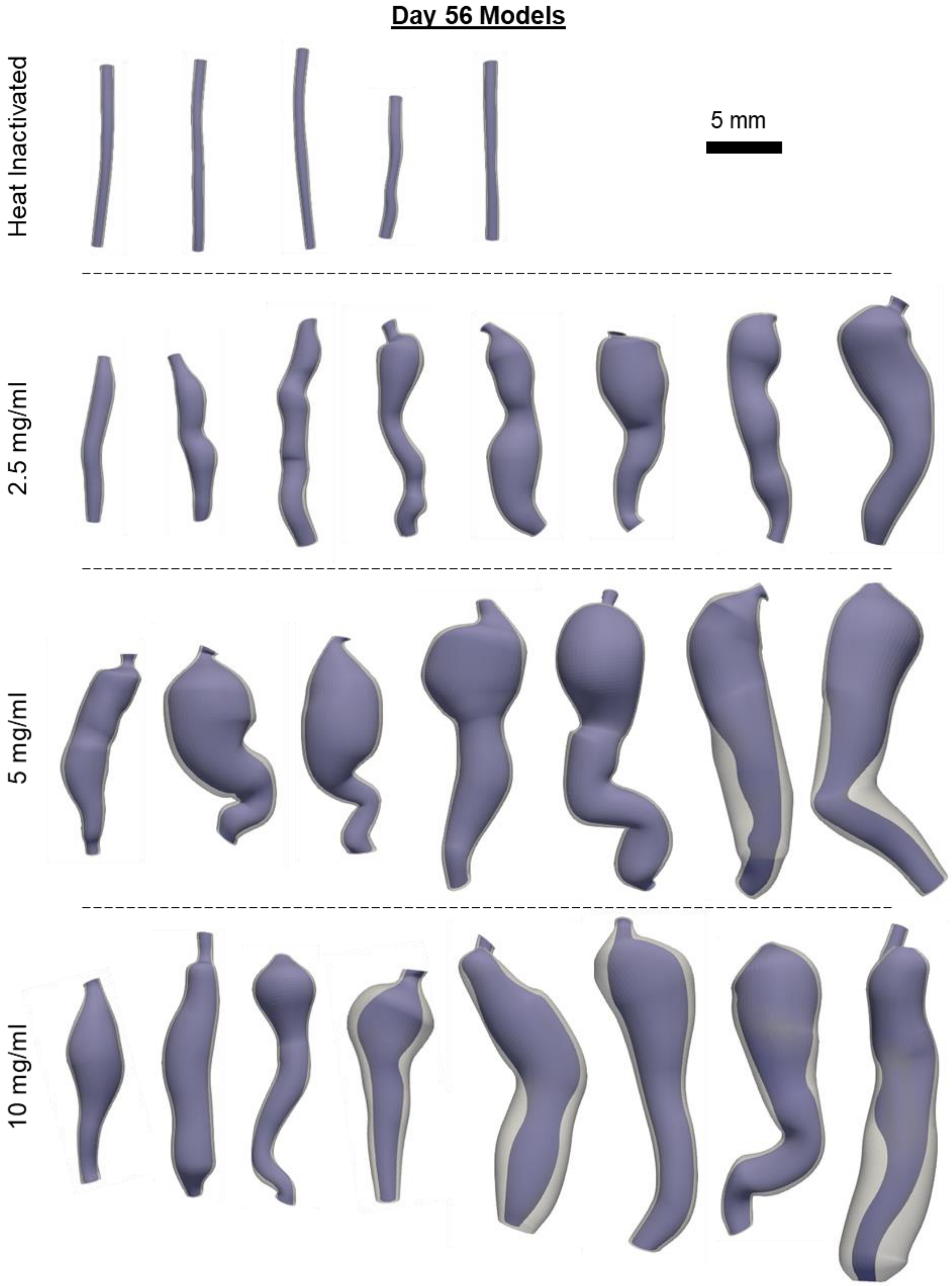

Supplementary Figure S3 – 3D ultrasound-based segmentations of mice 56 days post-surgery. Mice treated with 2.5 mg/ml generally appear smaller and lack the thrombus observed in the 5 mg/ml and 10 mg/ml groups. In the 5 mg/ml group, two mice had thrombus, which was lower than the five mice who had thrombus in the 10 mg/ml group, as indicated by the thicker walls in the segmentations.

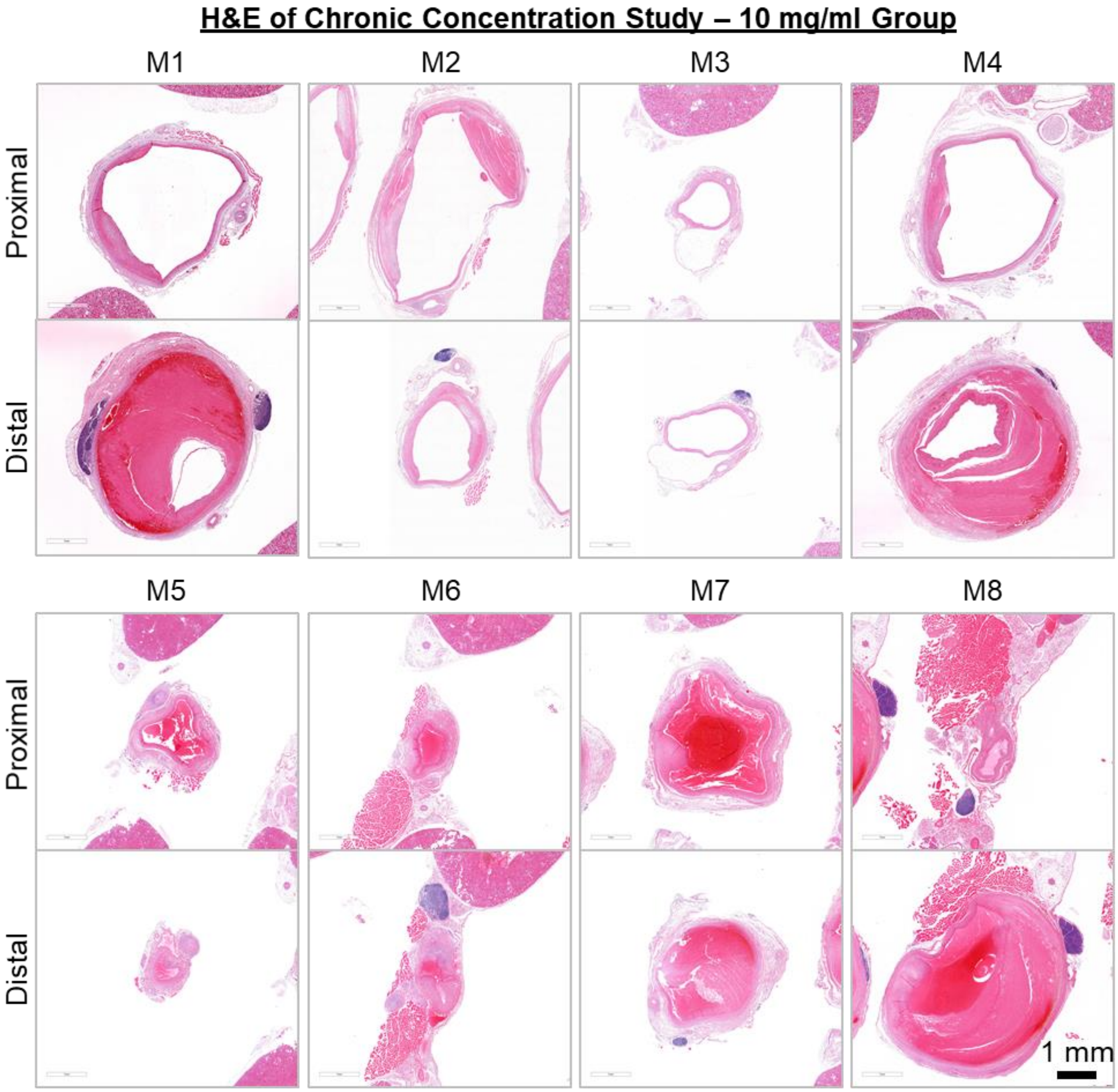

Supplementary Figure S4 – Hematoxylin and Eosin (H&E) staining of mice treated with 10 mg/ml elastase. Mice were euthanized 56 days post-surgery.

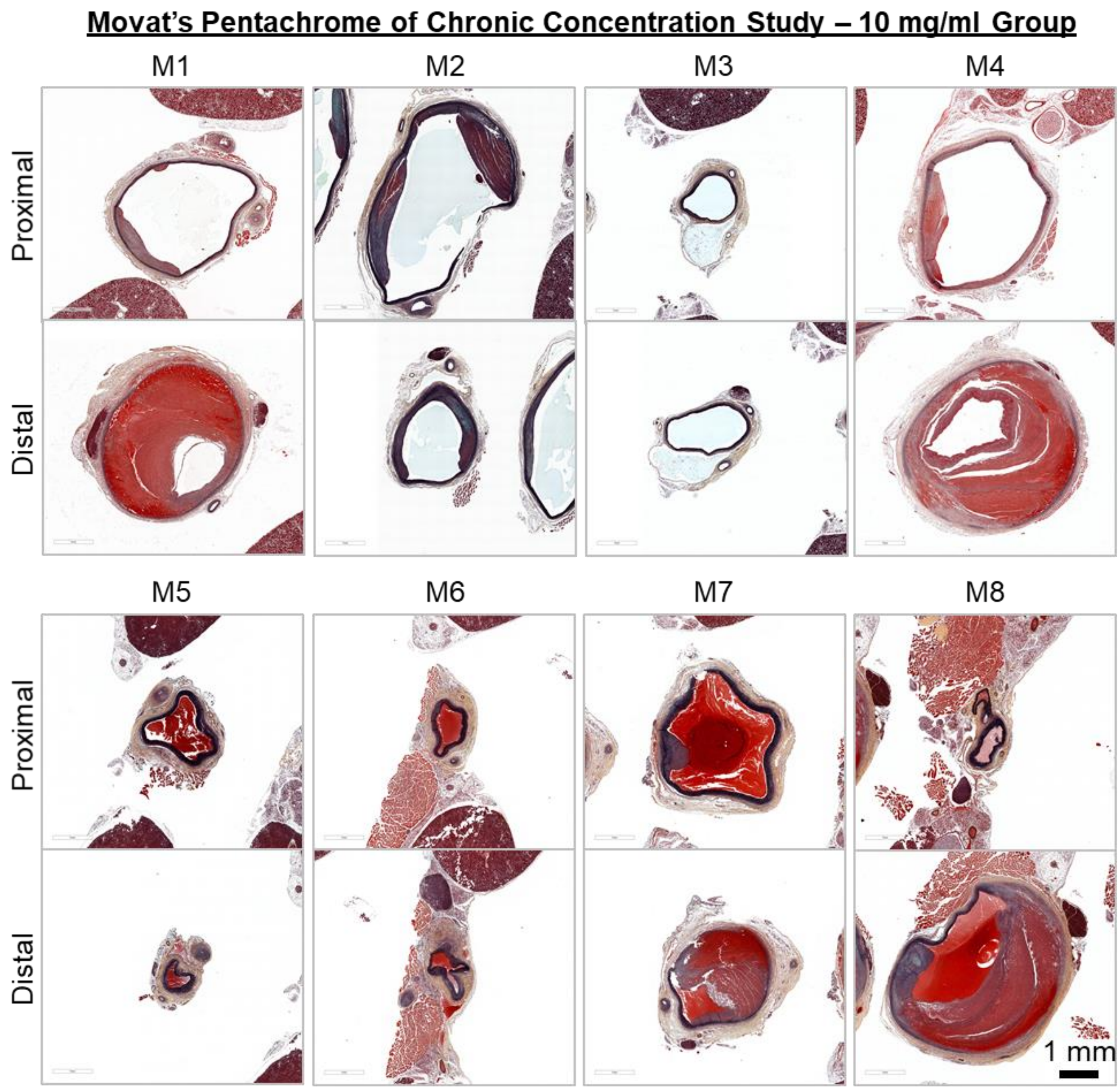

Supplementary Figure S5 – Movat's Pentachrome (MPC) staining of mice treated with 10 mg/ml elastase. Mice were euthanized 56 days post-surgery.

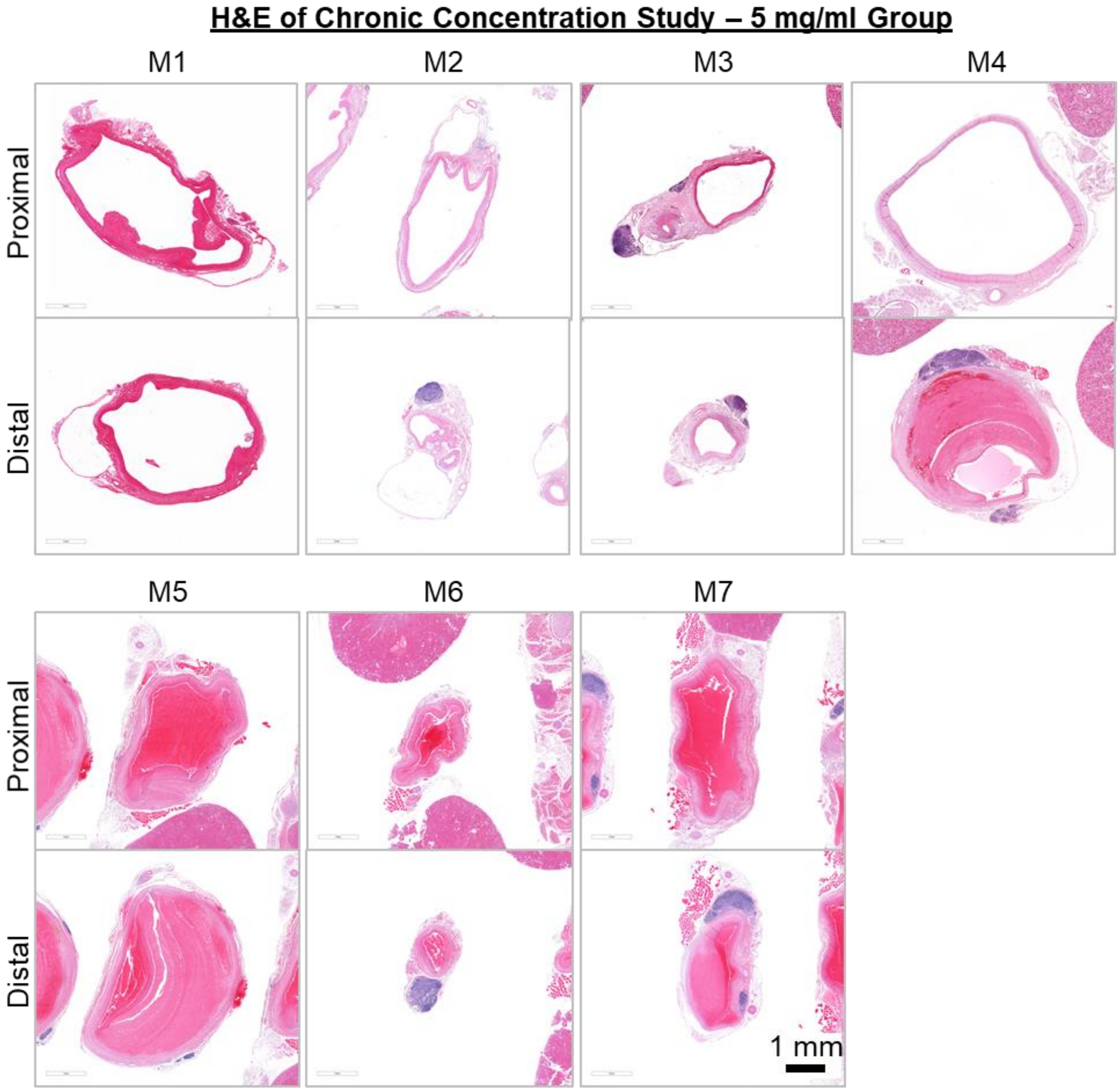

Supplementary Figure S6 – Hematoxylin and Eosin (H&E) staining of mice treated with 5 mg/ml elastase. Mice were euthanized 56 days post-surgery.

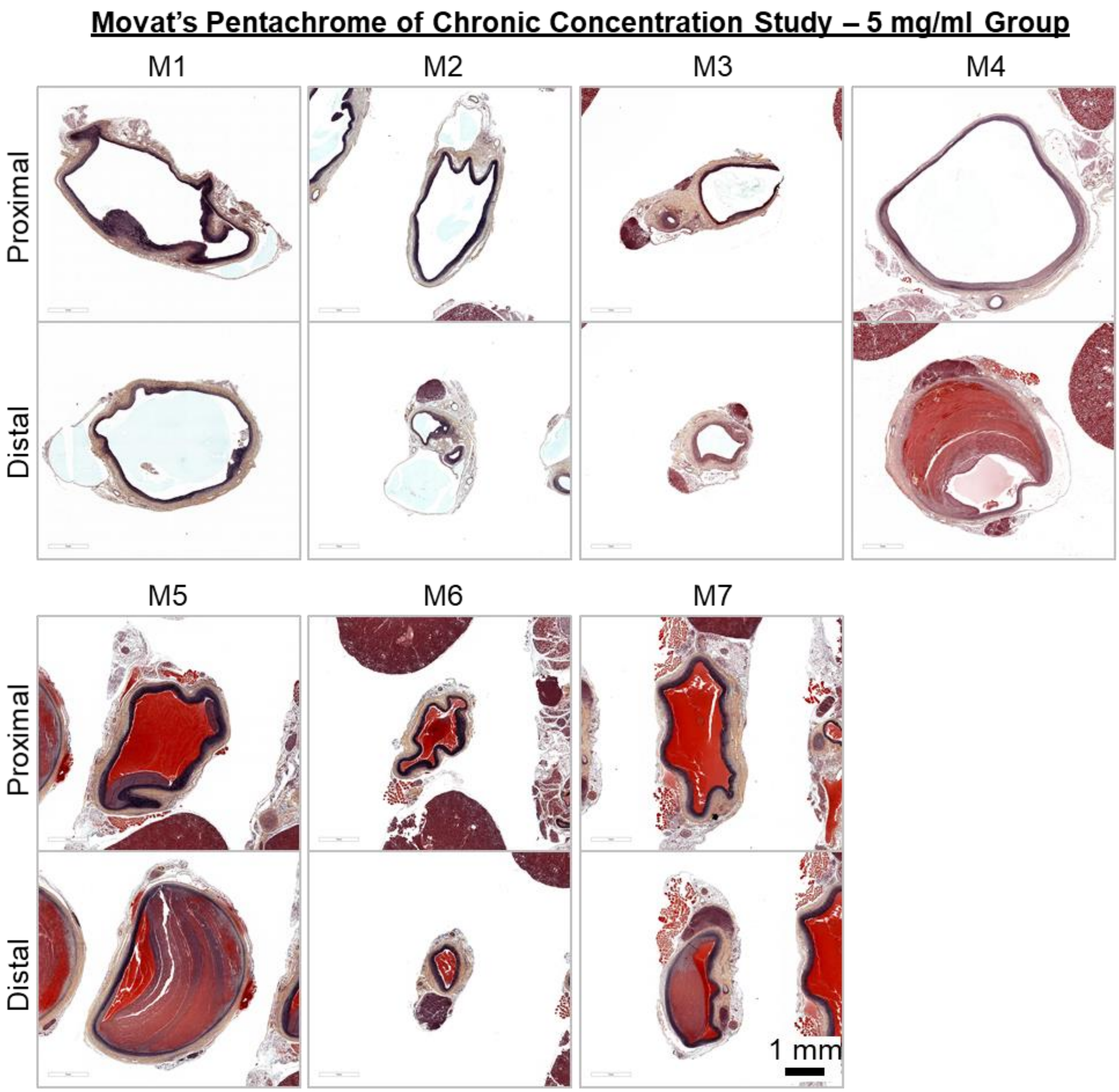

Supplementary Figure S7 – Movat's Pentachrome (MPC) staining of mice treated with 5 mg/ml elastase. Mice were euthanized 56 days post-surgery.

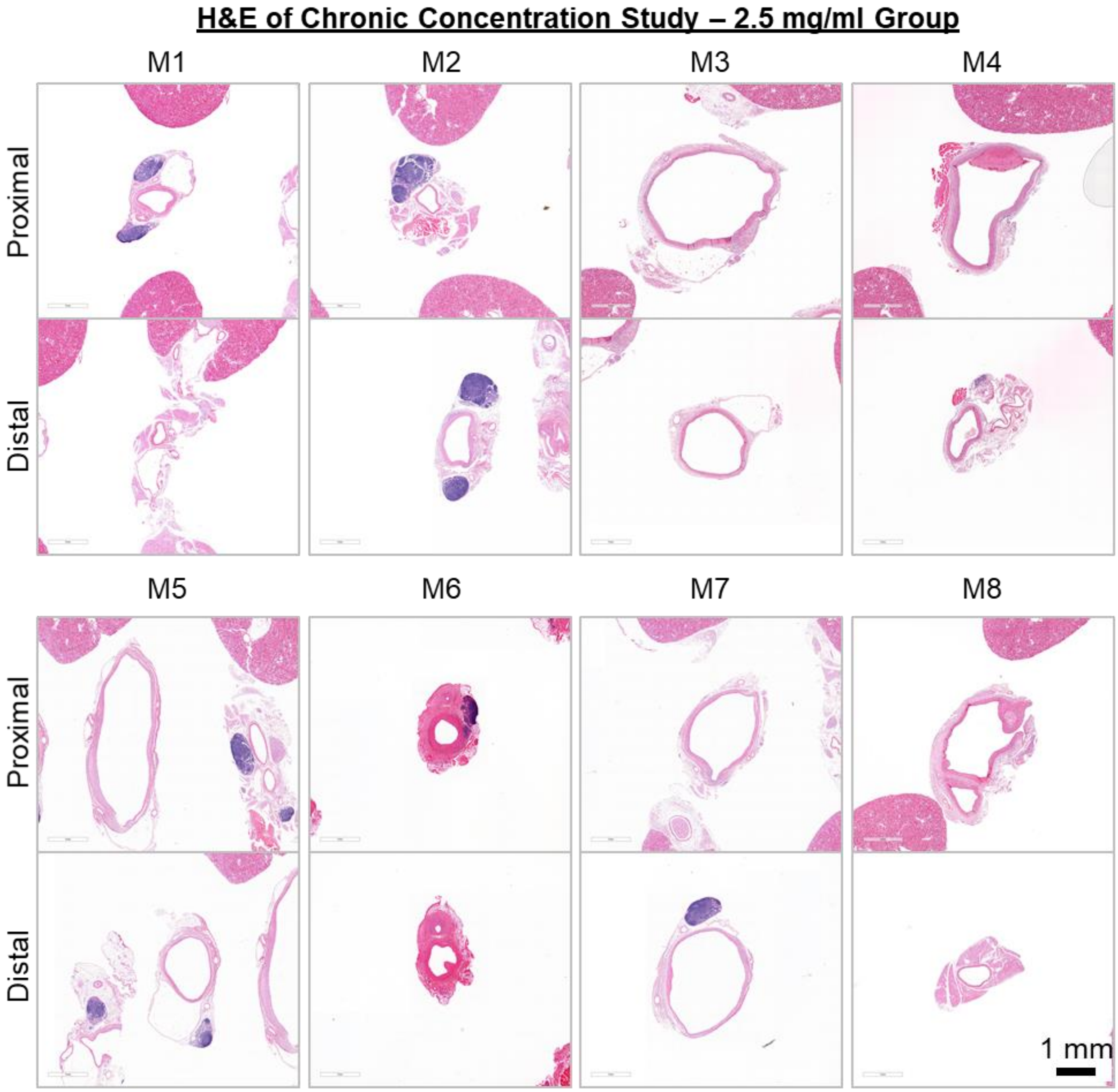

Supplementary Figure S8 – Hematoxylin and Eosin (H&E) staining of mice treated with 2.5 mg/ml elastase. Mice were euthanized 56 days post-surgery.

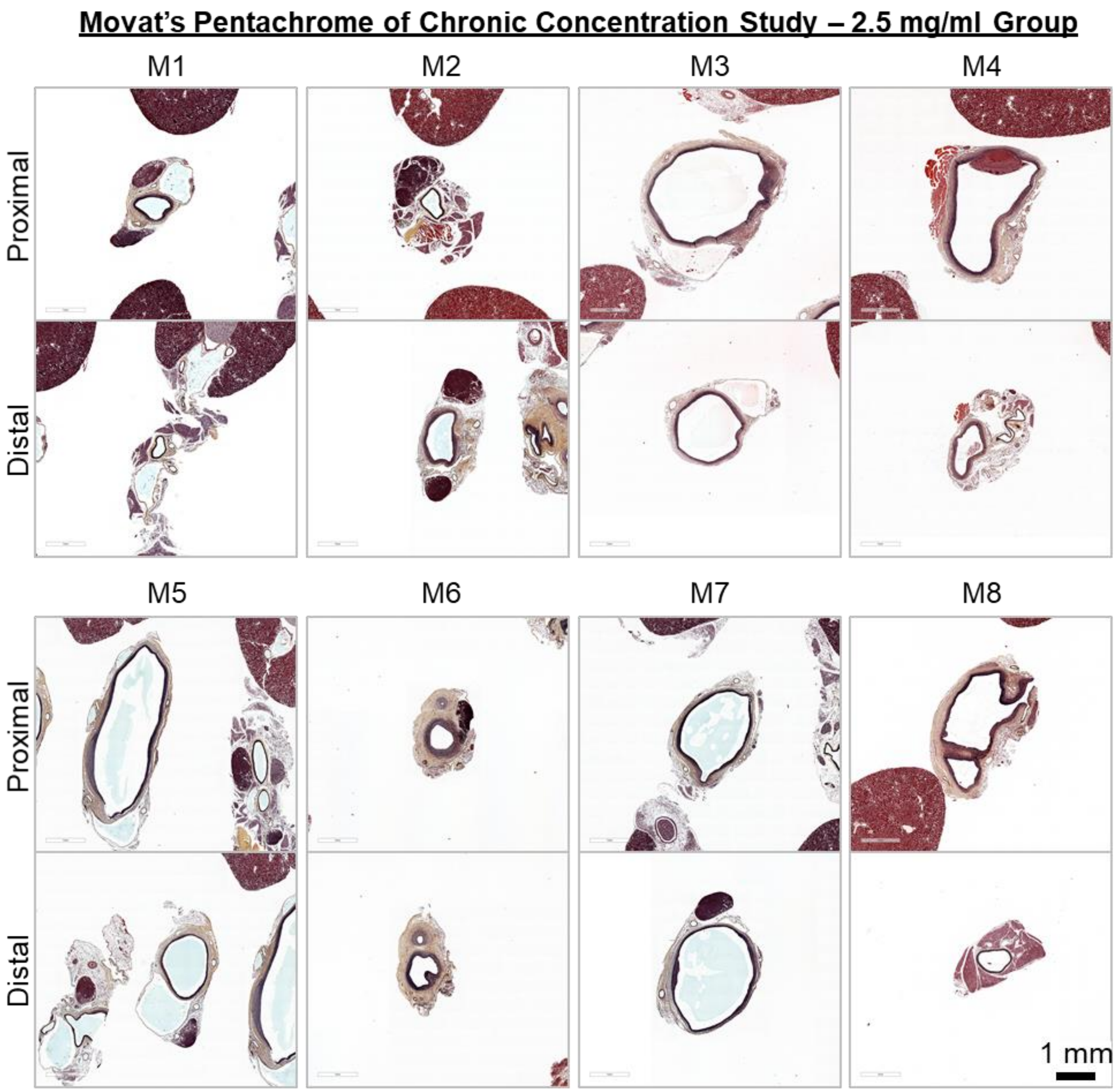

Supplementary Figure S9 – Movat's Pentachrome (MPC) staining of mice treated with 2.5 mg/ml elastase. Mice were euthanized 56 days post-surgery.

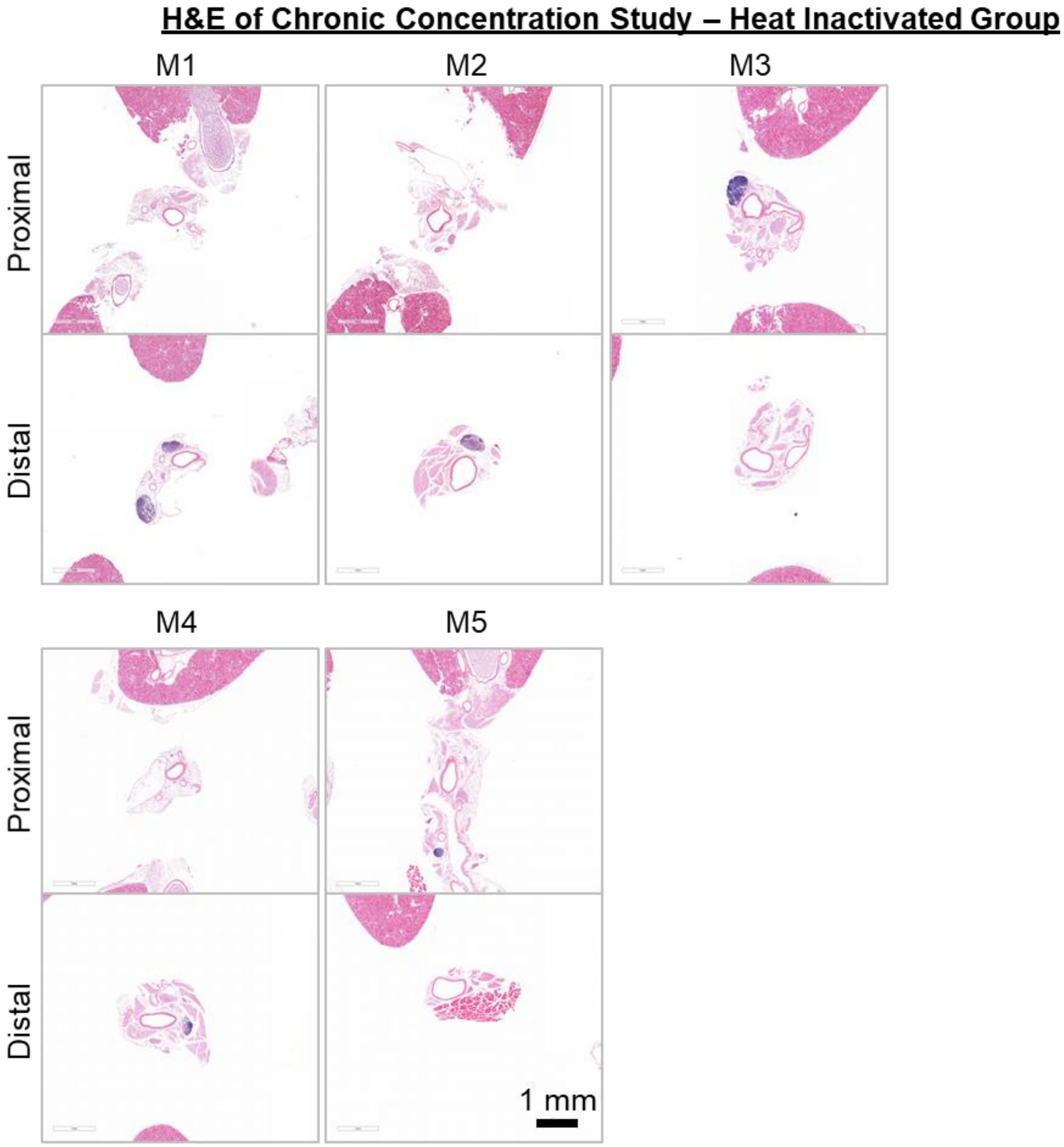

Supplementary Figure S10 – Hematoxylin and Eosin (H&E) staining of mice treated with heat-inactivated elastase. Mice were euthanized 56 days post-surgery.

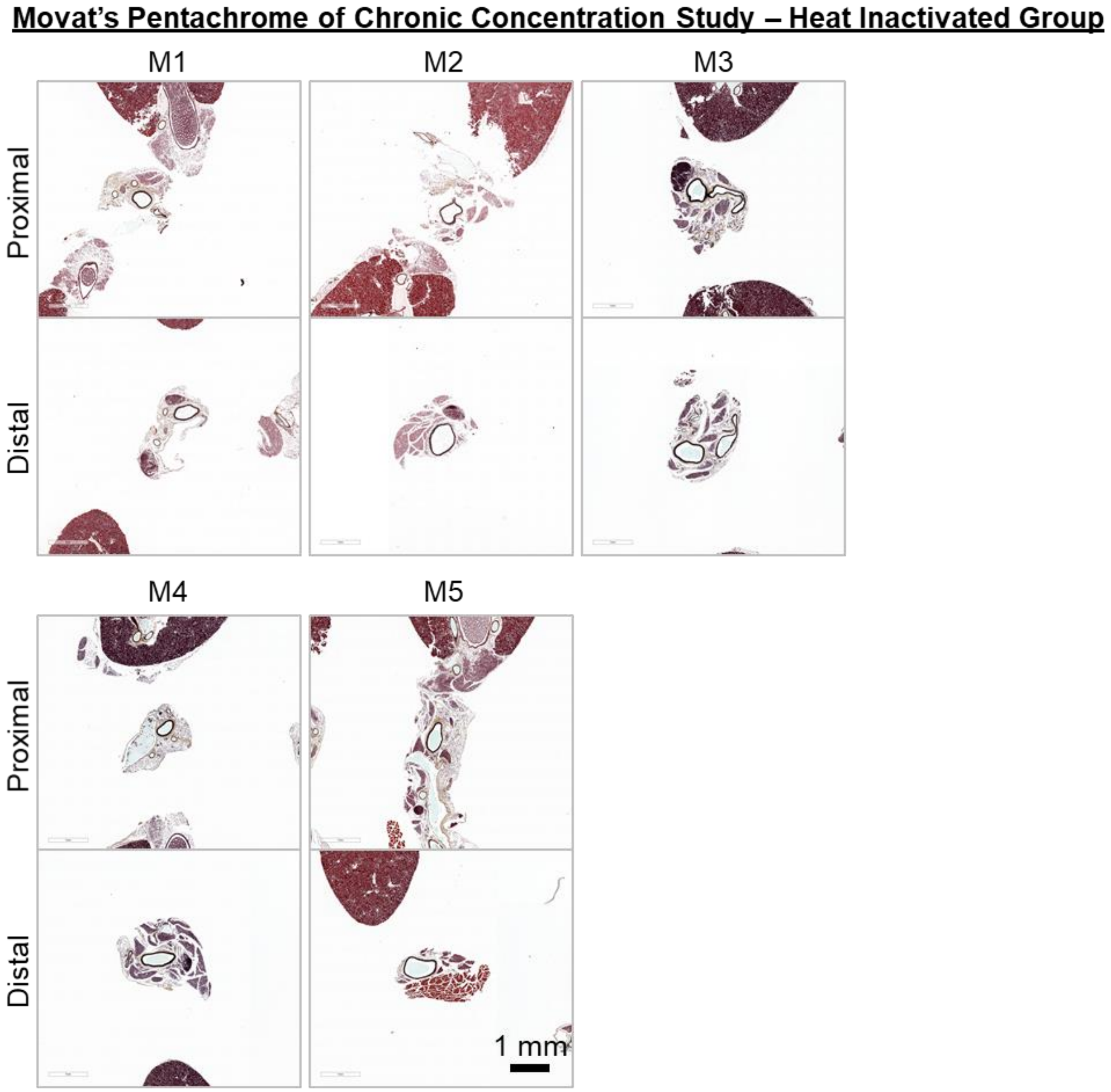

Supplementary Figure S11 – Movat's Pentachrome (MPC) staining of mice treated with heat-inactivated elastase. Mice were euthanized 56 days post-surgery.

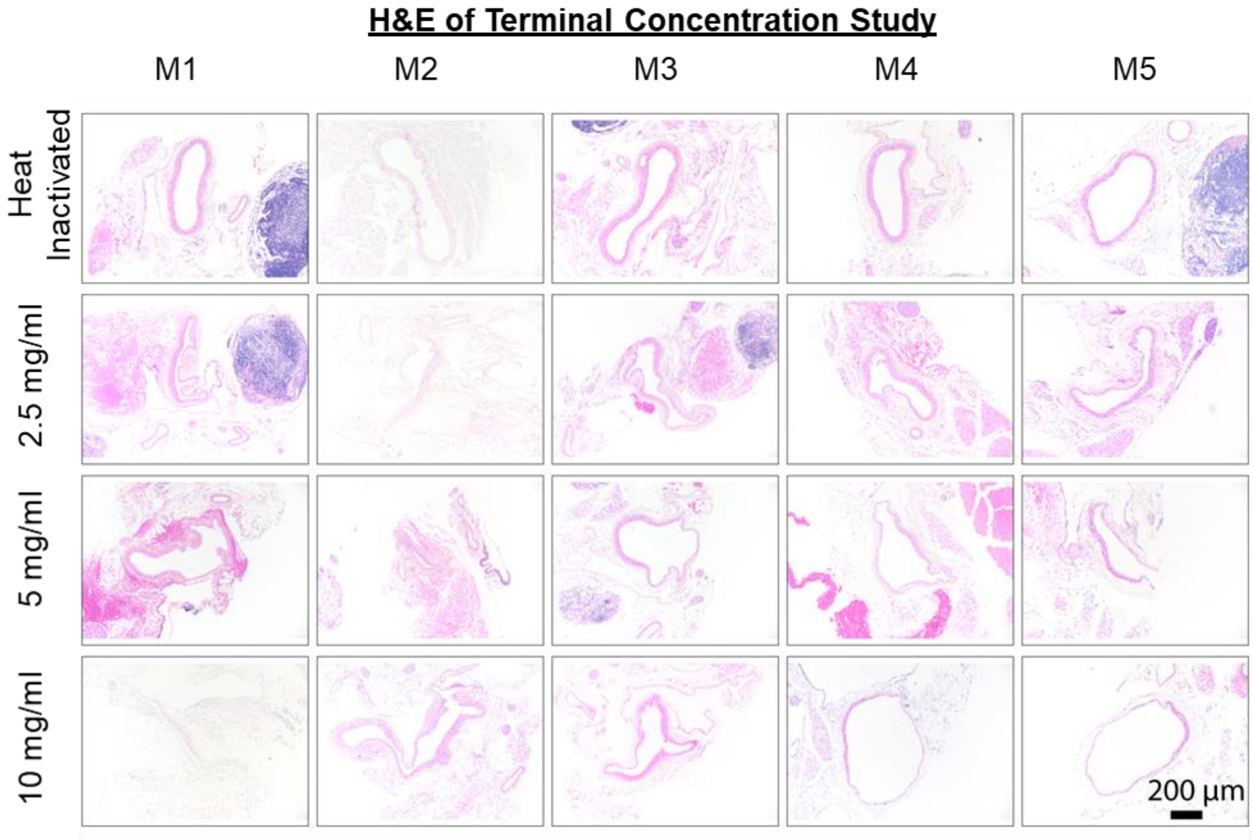

Supplementary Figure S12 – Hematoxylin and Eosin (H&E) staining of mice euthanized immediately post-surgery. The concentrations are of elastase applied to the aorta.

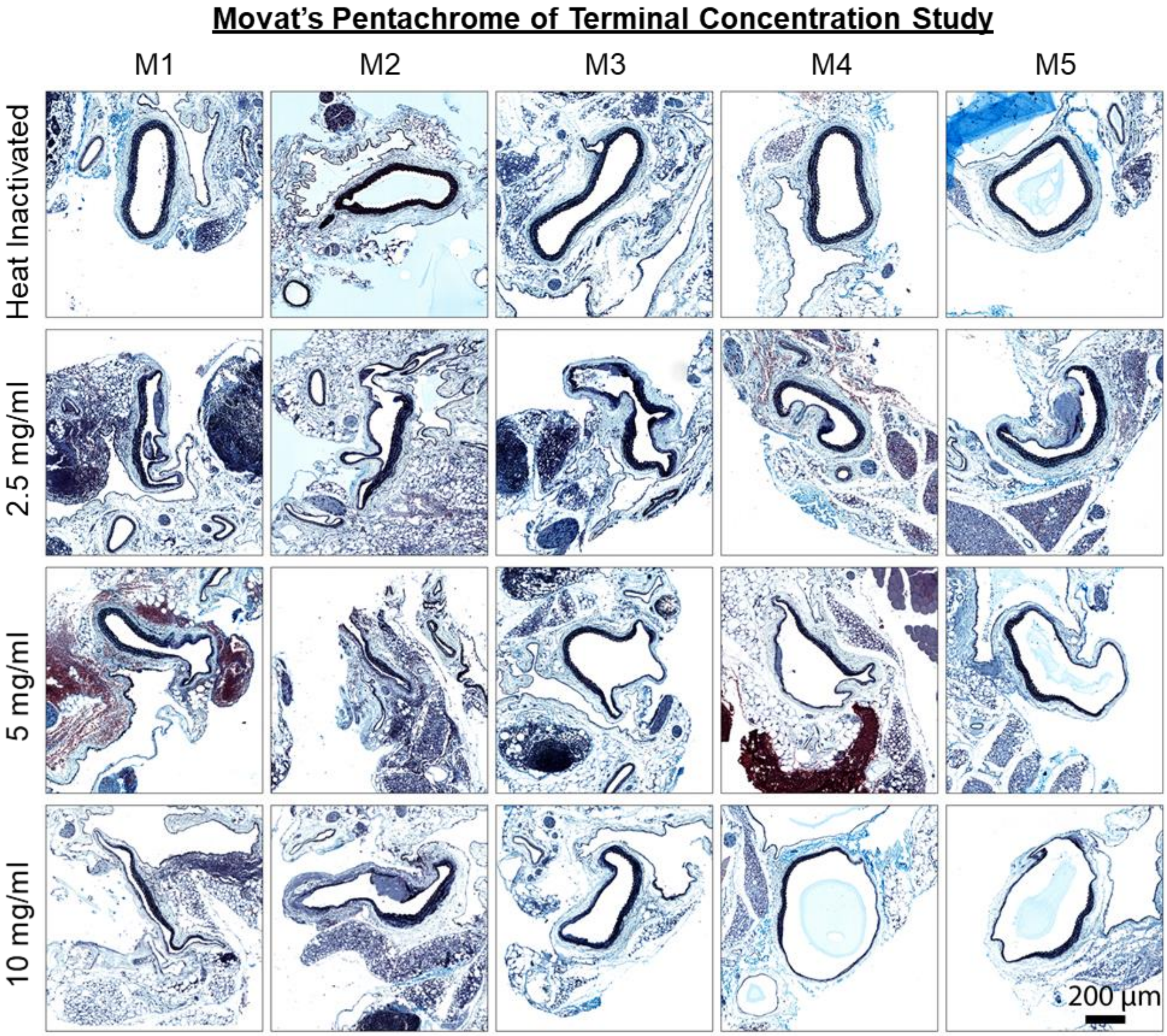

Supplementary Figure S13 – Movat's Pentachrome (MPC) staining of mice euthanized immediately post-surgery. The concentrations are of elastase applied to the aorta.

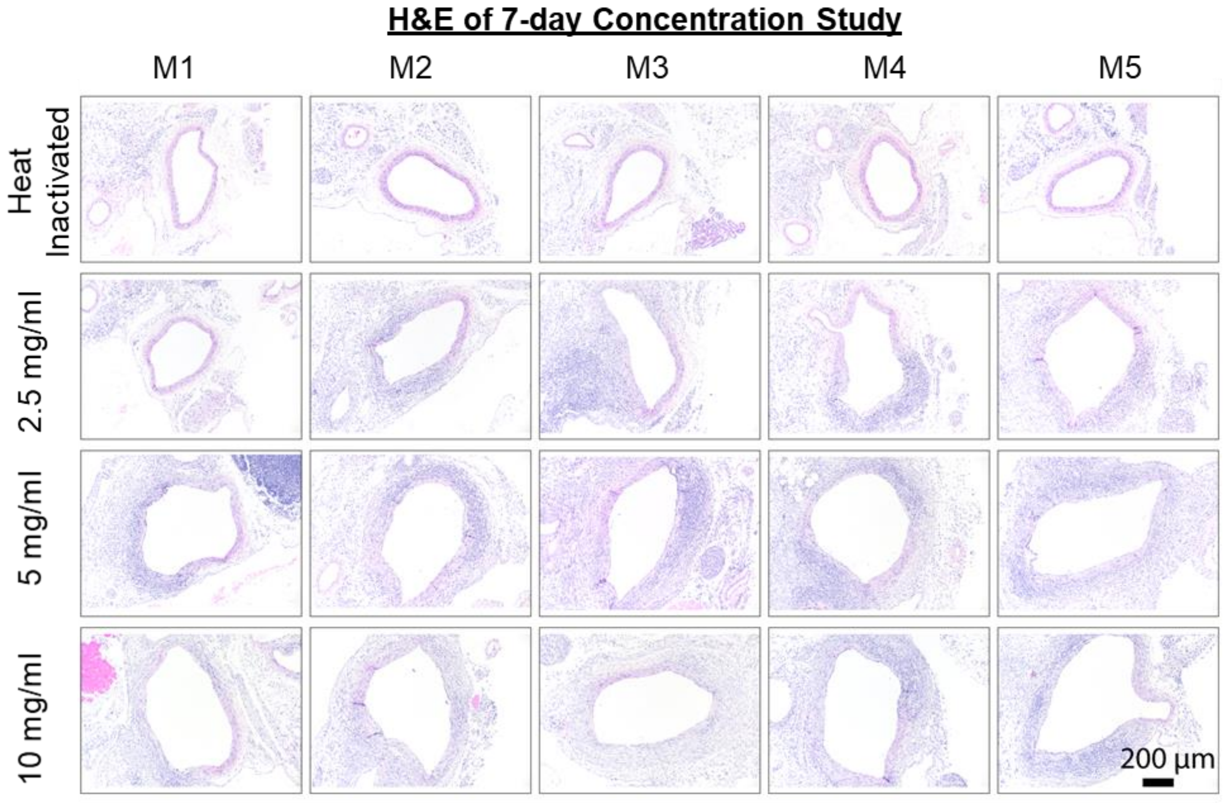

Supplementary Figure S14 – Hematoxylin and Eosin (H&E) staining of mice euthanized 7 days post-surgery. The concentrations are of elastase applied to the aorta.

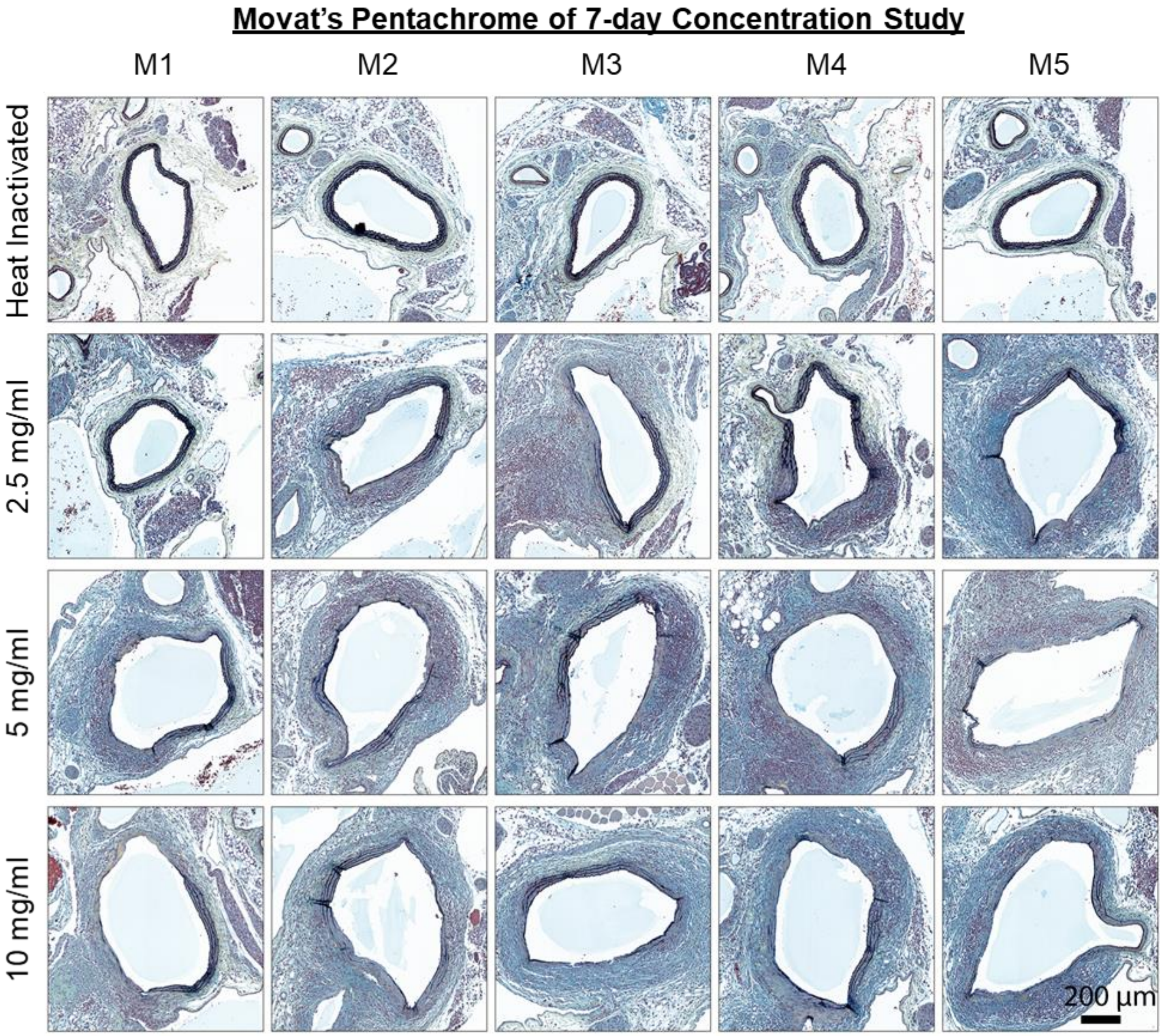

Supplementary Figure S15 – Movat's Pentachrome (MPC) staining of mice euthanized immediately post-surgery. The concentrations are of elastase applied to the aorta.

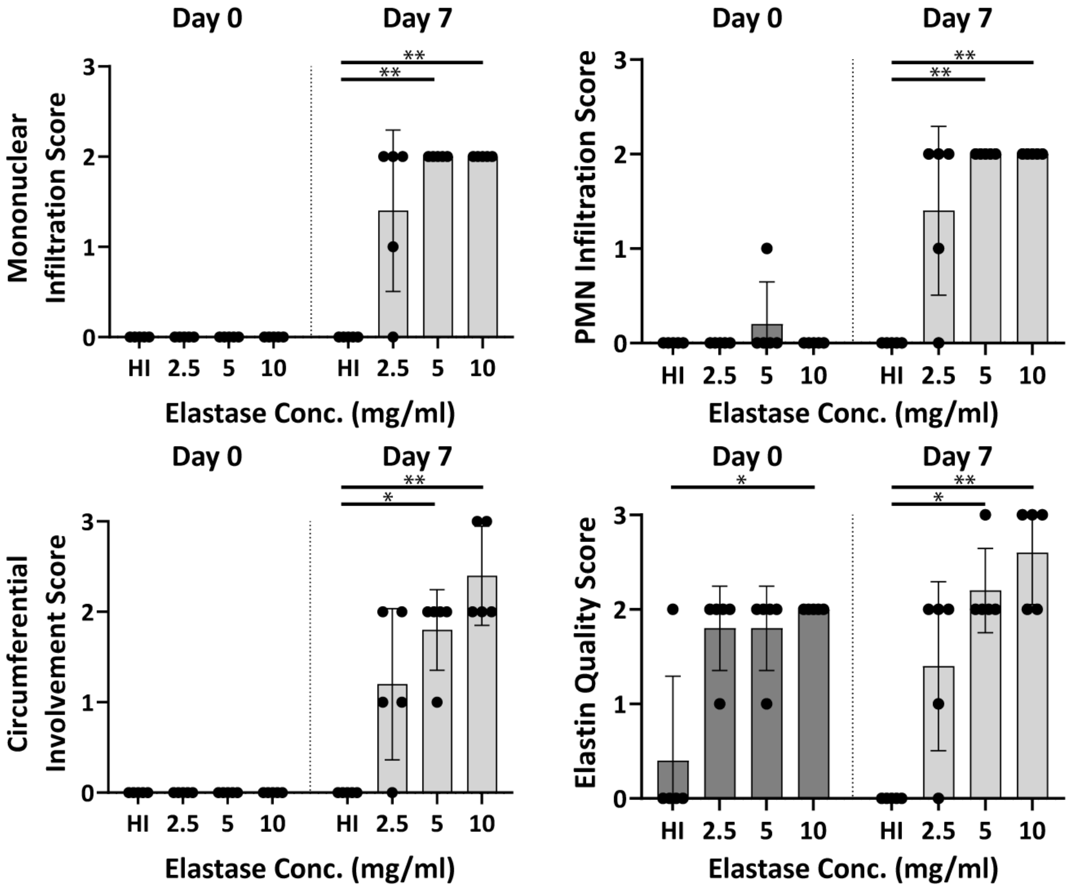

Supplementary Figure S16 – Semi-quantitative scoring of the H&E slides indicated mononuclear and polymorphonuclear leukocytes infiltration in the elastase-treated mice by day 7. Additionally, the infiltration was spread throughout more of the circumference of the aorta in the 5 and 10 mg/ml groups. Lastly, elastin quality was immediately reduced post-surgery, which continued through day 7. Due to the non-parametric nature of the scoring, a Kruskal-Wallis test was performed within each time point with a post-hoc Dunn’s test. *$p<0.05$, $^{**}p<0.01$*

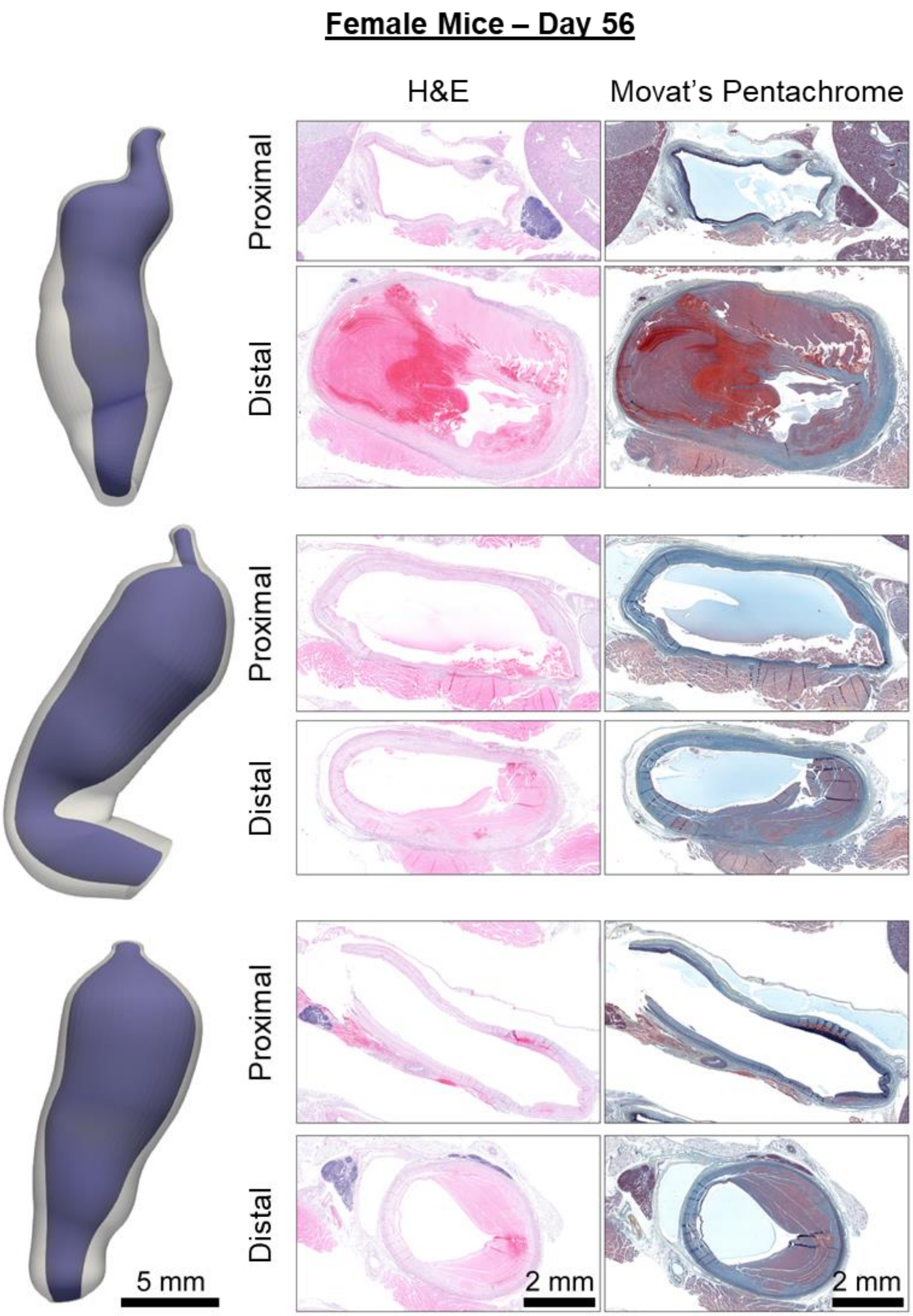

Supplementary Figure S17 – 3D ultrasound-based segmentations of female mice 56 days post-surgery. The aneurysms in these female mice were larger than that of the males at the same timepoint, and all female mice developed thrombus. Also shown is Hematoxylin and Eosin (H&E) and Movat's Pentachrome (MPC) staining in the proximal and distal sections.

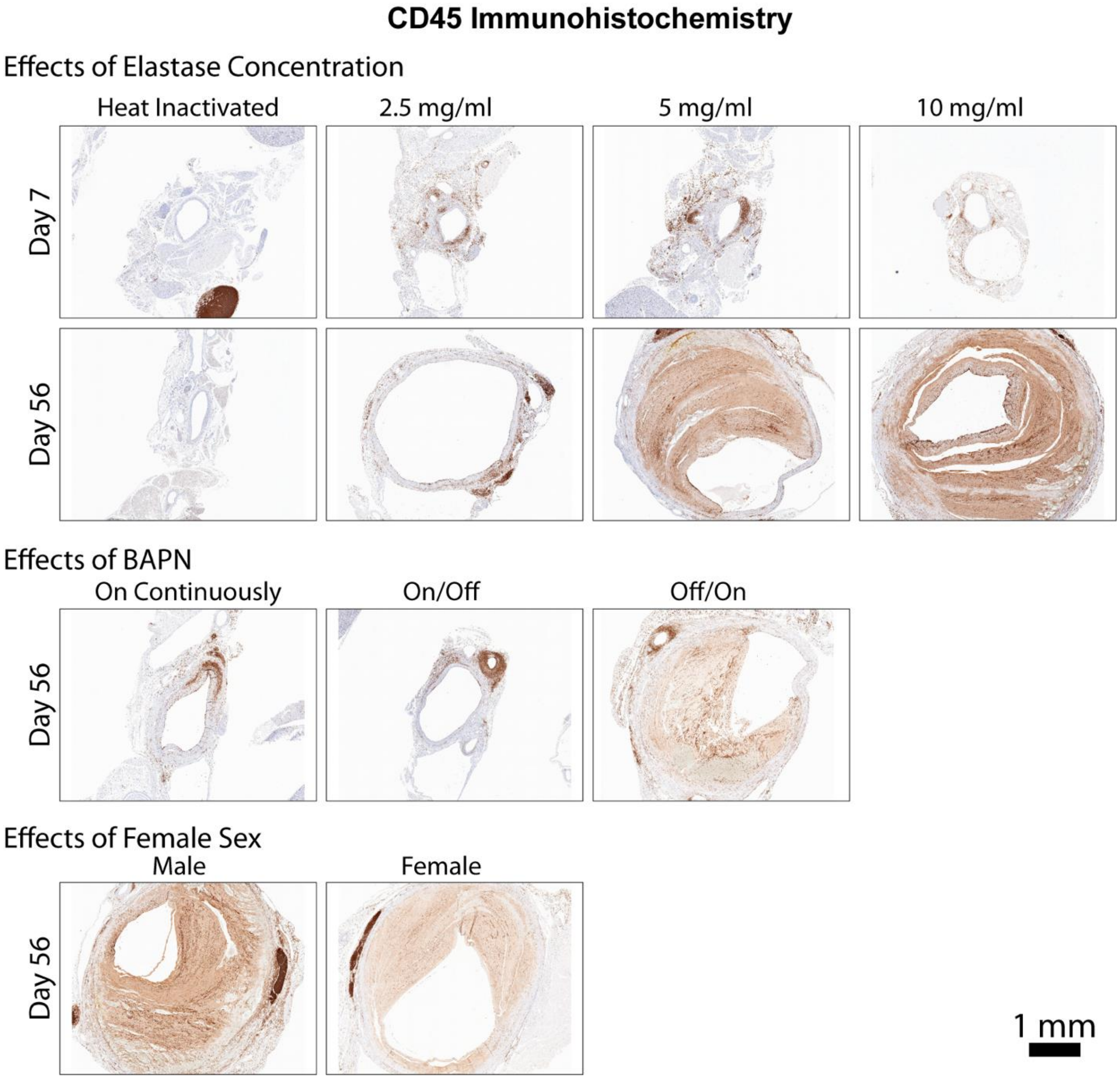

Supplementary Figure S18 – Example anti-CD45 immunohistochemistry (immunoperoxidase-DAB, hematoxylin counterstain) images of aortic tissue from mice euthanized days 7 and 56 post-surgery with varying levels of elastase, modifying the BAPN treatment, and in both male and female mice.

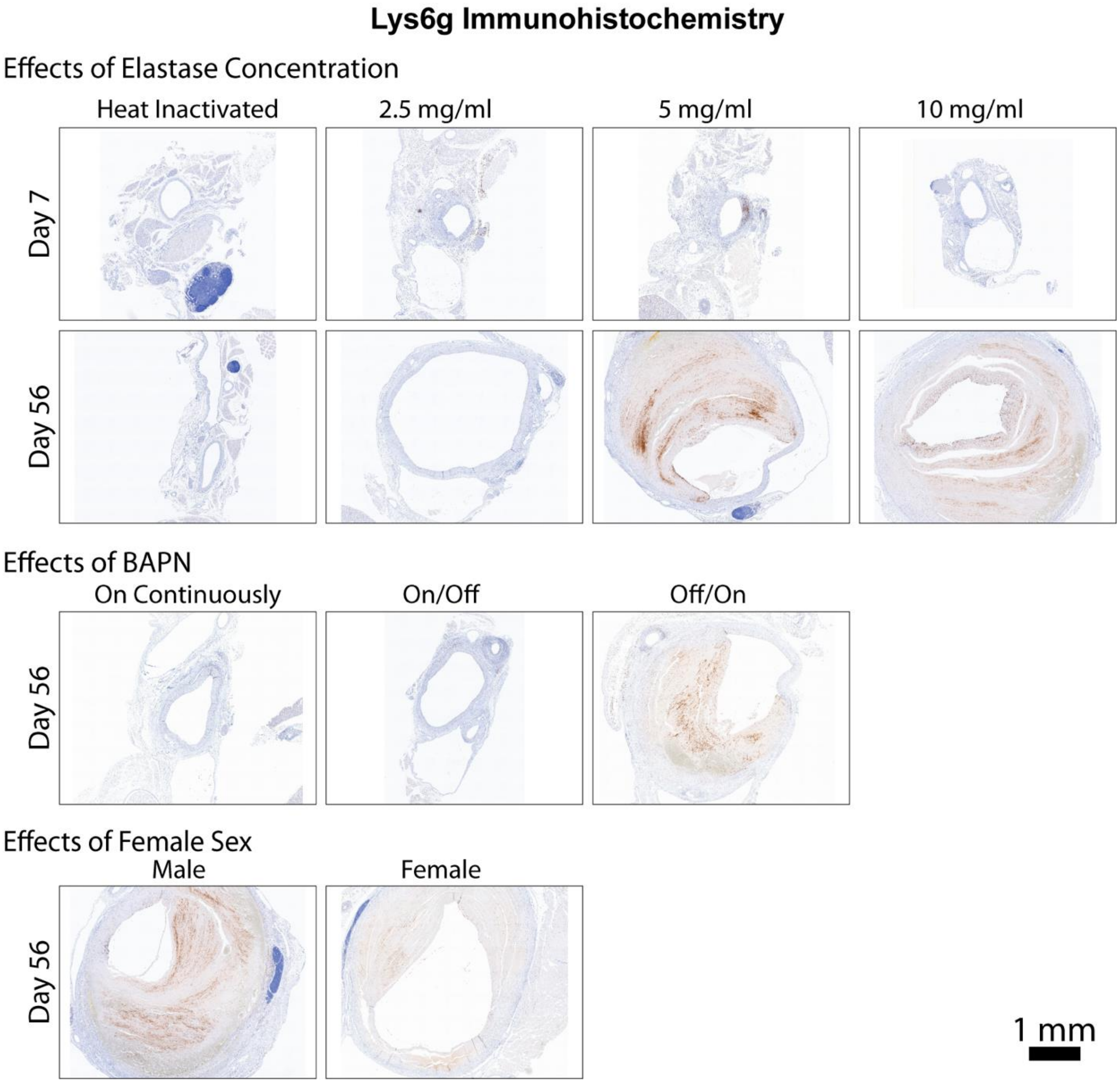

Supplementary Figure S19 - Example Lys6g immunohistochemistry (immunoperoxidase-DAB, hematoxylin counterstain) images of aortic tissue from mice euthanized days 7 and 56 post-surgery with varying levels of elastase, modifying the BAPN treatment, and in both male and female mice.

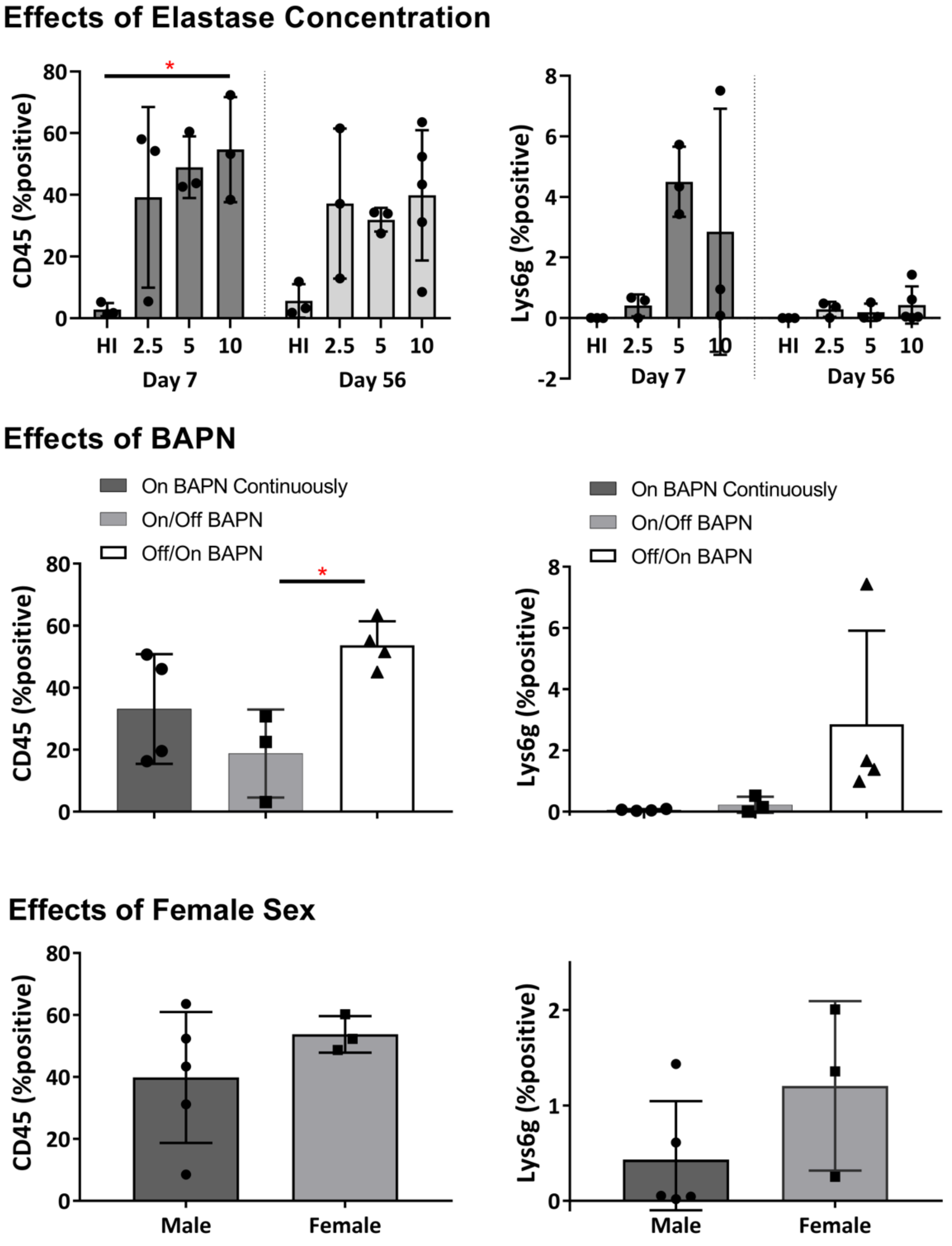

Supplementary Figure S20 - The aortas from a subset of mice were stained to assess immune cells (CD45) and neutrophils (Lys6g) in control and aneurysmal tissue. The control tissue consistently had a relatively low amount of immune cell infiltrates. Conversely, although it was often not significant in all cases, the aneurysmal tissues tended to have a higher number of inflammatory cells (*$*p<0.05$*). This inflammatory response matches the growth trends observed in the on/off and off/on BAPN groups. Similarly, female mice had higher amounts of immune cell infiltration and neutrophils compared to male animals, although this increase was not significant.

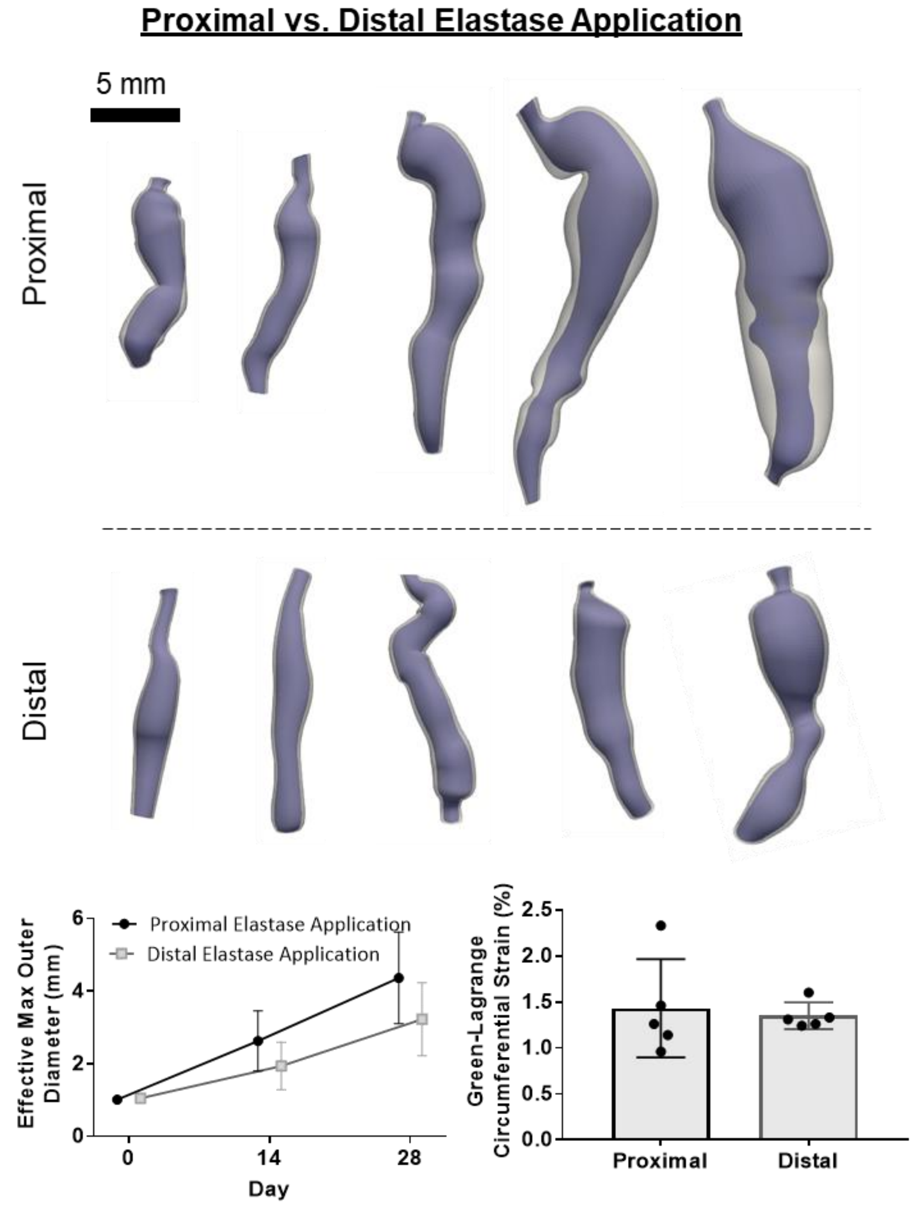

Supplementary Figure S21 – 3D ultrasound-based segmentations demonstrating the effects of applying elastase more proximally (i.e. closer to the renal arteries) compared to distally (i.e. closer to the trifurcation). Although diameter is not significantly different between groups, animals that received a proximal elastase application typically resulted in larger aneurysms.

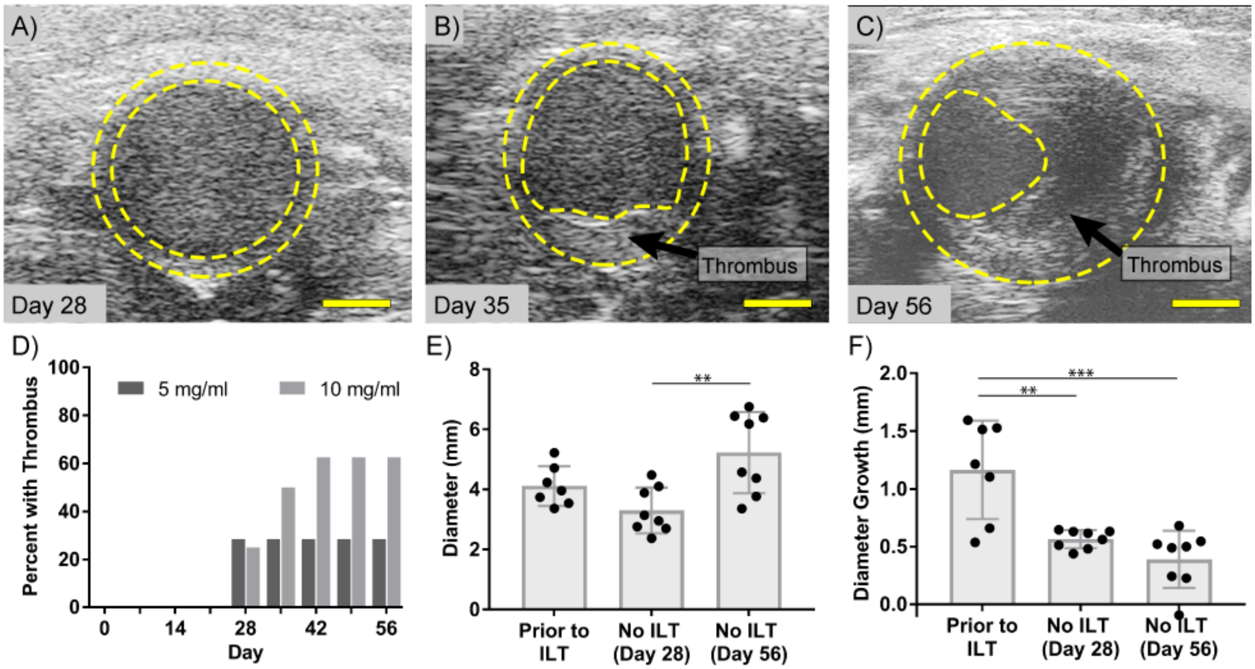

Supplementary Figure S22 – Presence of thrombus was noted in some of the 5 mg/ml and 10 mg/ml elastase-treated mice. A) Ultrasound images of a representative mouse show no thrombus on day 28, B) compared to day 35. C) Over time, the thrombus continued to grow and develop, resulting in a much higher thrombus burden by day 56. D) The percentage of mice with thrombus was greater in the 10 mg/ml group than the 5 mg/ml group, though results were non-significant. E) Combining mice in the 5 mg/ml and 10 mg/ml groups, we found that measurements of diameter from the week prior to observing thrombus were not significantly different from mice that had no thrombus at days 28 or 56. F) In contrast, mice that formed thrombus had a significantly higher diameter growth rate compared to mice that had no thrombus. Scale bars are 1 mm. *$p<0.05$; **$p<0.01$; ***$p<0.001$

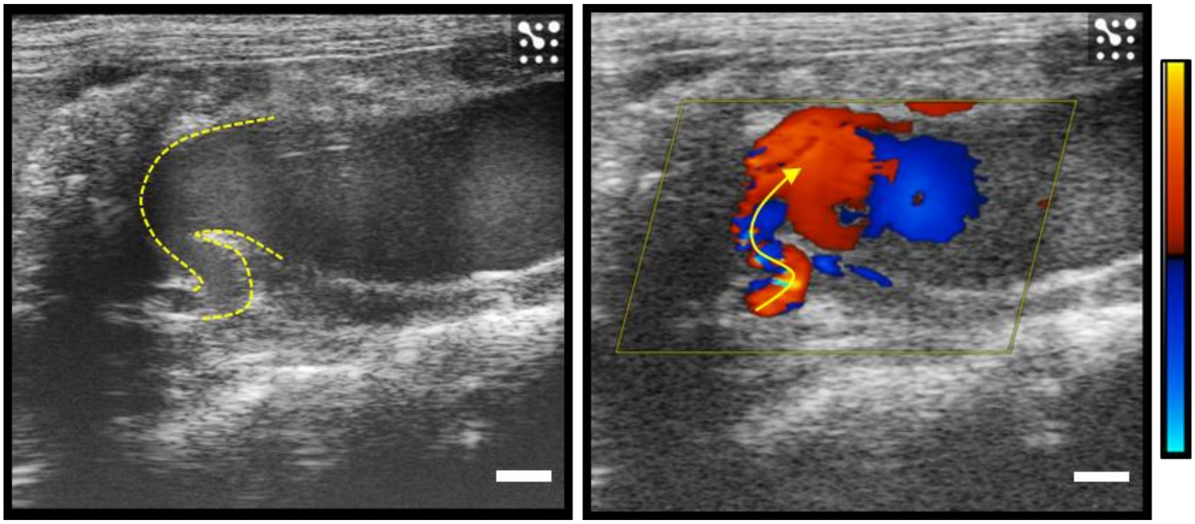

Supplementary Figure S23 – Long-axis EKV and Color Doppler images of a mouse the week prior to thrombus formation. Yellow dotted lines show the outline of the proximal portion of the vessel, while the yellow arrow indicates the path of blood flow. Overall, these images show a tortuous proximal non-aneurysmal region that causes a small jet inflow of blood into the abdominal aorta. Scale bar is 1 mm.

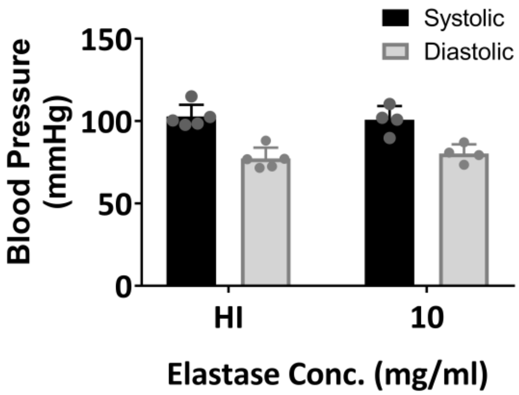

Supplementary Figure S24 – The blood pressure of 5 mice from the heat-inactivated and 4 mice from the 10 mg/ml groups were measured on Day 56. Results demonstrate that the mice were normotensive.
